# Computational Complementation: A Modelling Approach to Study Signalling Mechanisms during Legume Autoregulation of Nodulation

**DOI:** 10.1371/journal.pcbi.1000685

**Published:** 2010-02-26

**Authors:** Liqi Han, Jim Hanan, Peter M. Gresshoff

**Affiliations:** 1 ARC Centre of Excellence for Integrative Legume Research, The University of Queensland, Brisbane, Queensland, Australia; 2 School of Information Technology and Electrical Engineering, The University of Queensland, Brisbane, Queensland, Australia; 3 Centre for Biological Information Technology, The University of Queensland, Brisbane, Queensland, Australia; University of Washington, United States of America

## Abstract

Autoregulation of nodulation (AON) is a long-distance signalling regulatory system maintaining the balance of symbiotic nodulation in legume plants. However, the intricacy of internal signalling and absence of flux and biochemical data, are a bottleneck for investigation of AON. To address this, a new computational modelling approach called “Computational Complementation” has been developed. The main idea is to use functional-structural modelling to complement the deficiency of an empirical model of a loss-of-function (non-AON) mutant with hypothetical AON mechanisms. If computational complementation demonstrates a phenotype similar to the wild-type plant, the signalling hypothesis would be suggested as “reasonable”. Our initial case for application of this approach was to test whether or not wild-type soybean cotyledons provide the shoot-derived inhibitor (SDI) to regulate nodule progression. We predicted by computational complementation that the cotyledon is part of the shoot in terms of AON and that it produces the SDI signal, a result that was confirmed by reciprocal epicotyl-and-hypocotyl grafting in a real-plant experiment. This application demonstrates the feasibility of computational complementation and shows its usefulness for applications where real-plant experimentation is either difficult or impossible.

## Author Summary

Endogenous signals, such as phytohormones, play a vital role in plant development and function, controlling processes such as flowering, branching, disease response, and nodulation. However, the signalling mechanisms are so subtle and so complex that details about them remain largely unknown. In this study, we develop a “Computational Complementation” approach for the investigation of long-distance signalling networks during legume autoregulation of nodulation (AON). The key idea is to use computational modelling to complement the deficiency of an empirical model of an AON deficient mutant with hypothesised AON components. If the complementation restores a wild-type nodulation phenotype, the modelled hypotheses would be supported as reasonable. To evaluate the feasibility of this approach, we tested whether wild-type soybean cotyledons participate in AON, commonly controlled by “real” leaves. The test gave an affirmative result (i.e., cotyledons do have AON activity), which was subsequently confirmed by a graft experiment on real plants. Future applications of this approach may be to test candidate AON signals such as auxins, flavones, and CLE peptides, and other plant signalling networks.

## Introduction

Legumes are one of the largest families of flowering plants that occupy about 15% of Earth's arable surface; yet they provide 27% of the world's primary crop production and more than 35% of the world's processed vegetable oil [1], signifying their cropping potential. Legumes are also the major natural nitrogen-provider to the ecosystem, contributing roughly 200 million tons of nitrogen each year [2] equivalent to over 200 billion dollars worth of fertiliser replacement value. Underlying this powerful fixation capability is a plant developmental process termed “nodulation”, which results from the symbiosis of legume roots and soil-living bacteria broadly called rhizobia. Yet for a legume plant itself, excessive nodulation may cause over-consumption of metabolic resources and disproportional distribution of internal growth regulators [3], and may interfere with developmentally related lateral root inception and function.

Legume plants have evolved a long-distance systemic signalling regulatory system, known as autoregulation of nodulation (AON), to maintain the balance of nodule formation [3]–[7]. It has been hypothesised that the induction of the nodule primordium produces a translocatable signal Q, which moves through a root-shoot xylem pathway to the leaves. This Q signal, or an intermediate, is detected in the phloem parenchyma of leaf vascular tissue by a transmembrane leucine-rich repeat (LRR) receptor kinase [8] related in structure to CLAVATA1 in Arabidopsis. This kinase is referred to as *GmNARK* in soybean [9],[10], *HAR1* in Lotus [11], and *SUNN* in Medicago [12]. Q is presumed to be a CLV3/ESR-related (CLE) peptide [13],[14]. The perception of the Q signal by the LRR receptor kinase triggers production of a hypothetical shoot-derived inhibitor (SDI) that is transported to the root to inhibit further nodule initiation. SDI can be extracted from wild-type leaves, re-fed via petiole feeding into loss-of-function mutants, resulting in restoration of the wild-type phenotypes [15]. It is a small, water-soluble, heat-stable and inoculation-dependent molecule. However, other mechanisms involved in AON signalling remain largely unknown, though the pre-NARK events (those setting up the signal transmission and then Q signal transduction) as well as the post-NARK events (firstly KAPP phosphorylation, ensuing transcriptional changes, and then SDI production) are being investigated [10],[15],[16].

To help understand such biological complexities, system modelling has been broadly applied [17]–[19]. From a systematic view, behind the signalling mechanisms is a network of components connected by intricate interfaces, with activities such as “assembly, translocation, degradation, and channelling of chemical reactions” occurring simultaneously [20]. These components and their interactions – also responding to the temporally and spatially changing environment – frame dynamic and complex systems at multiple scales to orchestrate plant development and behaviour. As a full understanding of system properties emerging from component interactions cannot be achieved only by “drawing diagrams of their interconnections” [17], computational techniques become indispensable for processing massive datasets and simulating complex mechanisms [21].

Although computational approaches have been progressing rapidly for modelling plant signalling, such as for signal transport [22],[23], canalization [24] and signalling network [25], most efforts have focused on cellular or tissue levels. Since AON is in essence a long-distance inter-organ regulatory network, our investigation required modelling at the whole-plant scale. Functional-structural plant models [26], such as those developed for resource allocation [27]–[29] and shoot signalling [30]–[34], can take inter-organ communication into account and use plant architecture as a direct reporter of underlying processes. Functional-structural modelling allowed us to simulate the hypothesised AON signalling and integrate it with nodulation. Yet the major difficulty was not how to model the hypotheses but how to test them through modelling. To meet this challenge, we have developed a new approach – Computational Complementation – for AON study.

Following description of the computational complementation method, we will present its first application in investigating whether wild-type cotyledons participate as an SDI producer in the AON system. Previous studies have indicated that mRNA for *GmNARK*, which, if translated, is responsible for perceiving the Q signal and triggering the SDI signal, exists in wild-type unifoliate and trifoliate leaves. It is expressed in all vascular tissue [8] of the plant (including the root), but its product is functional only as a nodulation control receptor in the leaf [35]. Thus the RNA expression pattern does not match biological function in AON. Relevant to the investigation here, the vasculature of the cotyledon also expresses RNA for *GmNARK*; whether this is functional in AON signalling was unclear. Therefore we used computational complementation to test two opposing hypotheses: (a) cotyledons function as part of the root, incapable of perceiving Q and producing SDI; or (b) cotyledons function as part of the shoot, involved in regulating root nodules.

## Methods

Genetic complementation [36] is a classical approach to define genetic cause-and-effect relations. For example, assuming two mutant organisms exhibit the same phenotype caused by loss-of-function (recessive) mutations, then their hybrid will be wild-type, if the mutations are in different genes (called cistrons); conversely the hybrid will be mutant if the mutations are in the same cistron. In other words, the wild-type (functional) allele complements the deficiencies of the mutant. Genetic complementation is also used in transgenic analysis of organisms, as a loss-of function mutation in a candidate wild-type gene is deemed causal for a mutant phenotype if that mutant is effectively complemented by the transfer of a dominant wild-type allele. The complementation approach introduced here does not cross one genotype with another, but will use computational modelling to complement the deficiency (in an empirical model) of a mutant to determine if this recovers the virtual wild-type phenotype.

**Figure 1:**
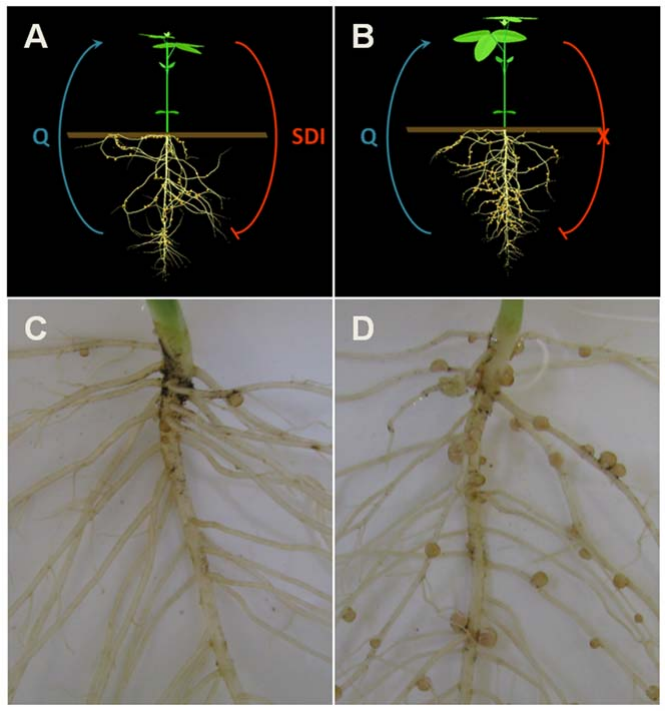
Wild-type soybean Bragg (left) and its supernodulation mutant *nts1116* (right). In the wild-type soybean Bragg, AON is well-established and the balance of nodulation is well maintained (A). This results in a phenotype with a normal number of nodules (C). In the mutant *nts1116*, GmNARK is not functional in the leaves, leading to the lack of SDI production (B) and consequently a supernodulation phenotype (D) with many more nodules than the wild type.

We use two well-characterized soybean (*Glycine max* L. Merrill) genotypes: the wild-type soybean Bragg and its loss-of-function mutant *nts1116* [37]. Wild-type soybean Bragg performs AON to keep its nodulation balance well-maintained (Fig. 1*A and C*), leading to characteristic crown nodulation in upper root portions. In its near-isogenic mutant *nts1116*, the Q signal generated from early nodule proliferation cannot induce SDI due to the lack of *GmNARK* activity in leaves (Fig. 1*B*). Reduced SDI in *GmNARK-*deficient plants leads to a phenotype with many more nodules than wild-type, called “supernodulation” or “hypernodulation” (Fig. 1*D*) [5]. Compared with Bragg, the only deficiency of *nts1116* plants is the significantly reduced capacity of producing SDI.

**Figure 2:**
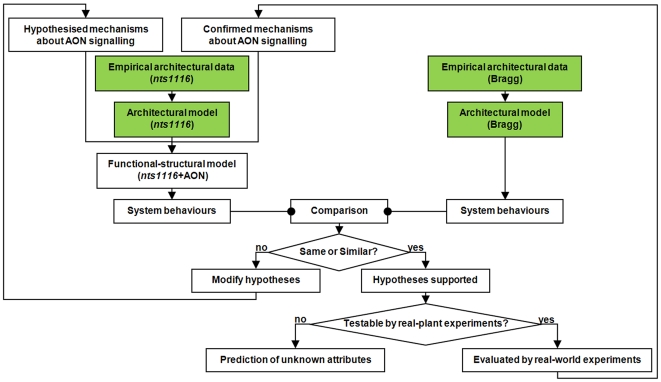
Flowchart of general computational modelling methodology. The first step (coloured in green) is to build empirical architectural models of parent cultivar Bragg and its derived mutant *nts1116*. The second step is to extend the *nts1116* architectural model to a functional-structural model enabling AON signalling. The confirmed and hypothesised mechanisms of AON signalling are then incorporated into the functional-structural model, to regulate the nodulation that cannot be regulated in real *nts1116* plants. The process is iterated until satisfactory comparison of system behaviours.

The key idea of our complementation approach comes from this point. We “add” hypothetical components of AON signalling, including those of signal production, transport, perception and function (see also Text S3), into the empirical model that depicts the growth behaviours of *nts1116* plants to see if a wild-type phenotype can be restored. The flowchart of methodology for this approach is given in Fig. 2, including the following steps:

- Build empirical models to simulate architectural development of Bragg and *nts1116* plants based on biometric growth data collected from cultivation of the two genotypes under the same conditions. The empirical data include architectural information such as internode length and diameter, petiole length and diameter, leaf length and width, lateral root branching patterns, and nodule number and distribution. Based on detailed organ-scale data, the architectural model can output realistic and dynamic visualisations as well as statistics of phenotypic development at a whole-plant scale. We call these outputs “system behaviours”.
- Extend the architectural model of a *nts1116* plant to a functional-structural model where simulation of inter-organ signalling activities is enabled and integrated with the signalling-development processes.
- Parameterise the functional-structural model built in step (ii) based on the confirmed and the hypothesised mechanisms about AON signalling. After this parameterisation, we call the functional-structural model “*nts1116*+AON”. The *nts1116*+AON model complements the deficiency of *nts1116*, and the resulting system behaviours represent a new nodulation phenotype.
- Compare the new phenotype generated by the *nts1116*+AON model in step (iii) and the nodulation pattern produced by the Bragg architectural model in step (i). If they are same or similar, the hypotheses will be supported as reasonable. Otherwise, the hypotheses need to be modified and tested again from step (iii).
- If the hypotheses supported in step (iv) are testable by real-world experiments, the virtual-experiment process can suggest appropriate real-experiment methods to further evaluate them. The mechanisms further supported by real-plant experiments will then be used as “confirmed mechanisms” in step (iii) to serve the testing of remaining hypotheses.
- If the hypotheses supported in step (iv) are not suitable or possible for evaluation through real-world experiments (due to limitation of current biological techniques), unknown attributes or characteristics about AON signalling can be predicted by virtual experiments.

The architectural and functional-structural models mentioned in steps (i) and (ii) have been built with context-sensitive L-systems [31]. The empirical data used for building architectural models of Bragg and *nts1116* plants were collected every second day from growth experiment under the same conditions until the 16^th^ day post-sowing (all plants were inoculated on the 2^nd^ day). Materials and methods for this glasshouse experiment are given in supporting Text S1. The growth data, algorithms and techniques used for model construction are described in supporting Text S2. The remaining steps of the flowchart, including (iii), (iv), (v) and (vi), are implemented for hypotheses testing and prediction.

## Results

### Application of the Approach through Virtual Experiments

In this initial application of our computational complementation approach, two opposing hypotheses were tested: (a) cotyledons function as part of the root, incapable of perceiving Q and producing SDI (abbreviated as “cotyledon-root” hypothesis); (b) cotyledons function as part of the shoot, involved in regulating root nodules (abbreviated as “cotyledon-shoot” hypothesis). Since *GmNARK* is expressed in all organs [8] (including cotyledons) and since cotyledons are short-term terminal organs (as they are degraded 7–14 days after germination), neither the cotyledon-root nor the cotyledon-shoot hypothesis was favoured a priori.

Theoretically speaking, if all other AON mechanisms (such as signal production, transport, perception and function) had been confirmed and used as basis for this application, the tested hypothesis leading to a wild-type nodulation pattern could be the correct one. However, the actions of many other signalling components also remain unclear. One or two virtual experiments are obviously insufficient to allow conclusions. Implementing too many experiments (to test all mechanisms together), however, would miss the emphasis and undermine efficiency. With these concerns, our strategy was to adjust parameters for signal production, transport, perception and function within a limited range, and use them as different conditions for different virtual experiments. Among all these experiments, if the complementation results (*nts1116*+AON) based on the cotyledon-root hypothesis are always or in most cases closer to Bragg than those based on the cotyledon-shoot hypothesis, then the cotyledon-root hypothesis would be considered plausible; otherwise, the cotyledons are more likely to function as general-sense leaves to regulate root nodulation.

According to this specific strategy, 27 virtual experiments (varying three rates of transport for both Q and SDI and three levels of nodulation inhibitory threshold) were designed for each of the two hypotheses: CRH_1∼CRH_27 for cotyledon-root testing and CSH_1∼CSH_27 for cotyledon-shoot testing. The only difference between CRH_i and CSH_j, if i = j, is whether cotyledons can function for AON signalling or not. Details of the virtual-experiment assumptions and conditions are described in the supporting Text S3.

**Figure 3:**
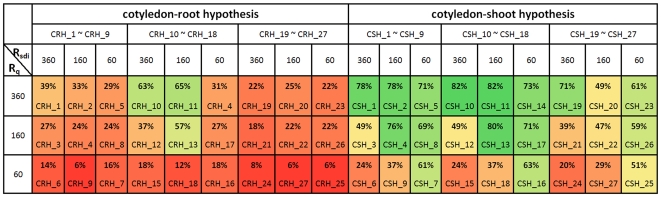
Complementation similarity degrees (10 days after sowing, 8 days after inoculation). The virtual-experiment results based on cotyledon-root hypothesis were all unsatisfactory on the 10^th^ day, while there were good results produced by cotyledon-shoot experiments. The colours varying from red to blue represent lower to higher similarity degrees (*cf.* Fig. 5).

**Figure 4:**
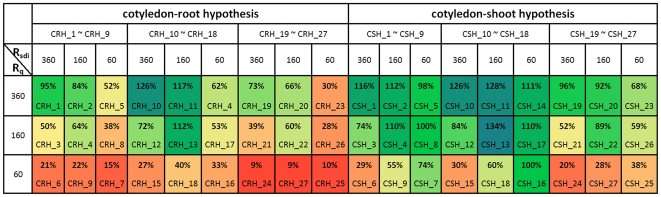
Complementation similarity degrees (16 days after sowing, 14 days after inoculation). On the 16^th^ day, four of the cotyledon-root experiments resulted in good similarity degrees, according to the criterion defined in Fig. 5. In comparison, there were twelve cotyledon-shoot experiments with good results produced.

**Figure 5:**
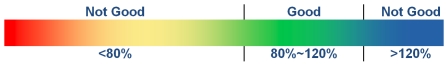
Criterion for evaluation of complementation similarity degree. If a similarity degree is between 80% and 120%, the complementation result it represents is viewed as “good”; otherwise the complementation result is viewed as “not good”.

**Figure 6:**
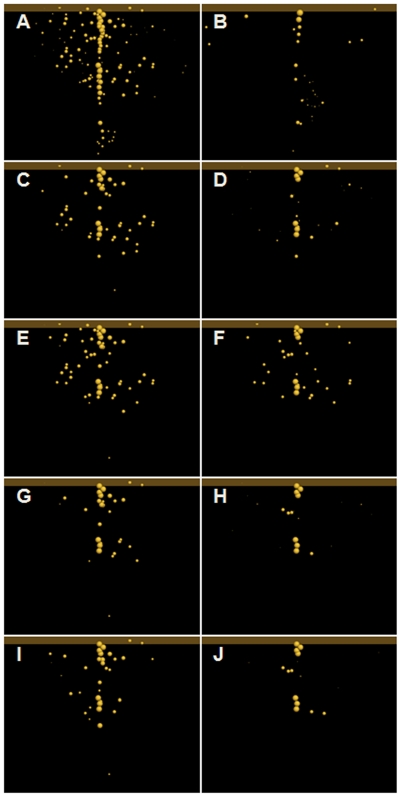
Visualisation of nodule distribution on the 16th day post-sowing. The primary and lateral roots were filtered in these visualisations to permit better observations of differences between nodule distribution patterns. As a guide, the pattern of yellow spots signifies essential AON characteristics in panel B, namely crown nodulation, restricted nodule number and small nodulation interval. (A) Nodule distribution generated by the *nts1116* architectural model. (B) Nodule distribution generated by the Bragg architectural model. The distribution patterns (C) (E) (G) and (I) resulted respectively from virtual experiments CRH_1, CRH_2, CRH_11 and CRH_13. The (D) (F) (H) and (J) were from CSH_1, CSH_2, CSH_11 and CSH_13. Potential nodules, which were not formed because of inhibition, can also be made visible, as shown by supporting Figure S1.

To quantify the comparison between complementation results and Bragg phenotype, we define their similarity degree *S_cp_* as
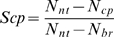
(1)where *N_nt_*, *N_br_* and *N_cp_* are the nodule numbers generated respectively by the architectural model of *nts1116* plants, the architectural model of Bragg, and the functional-structural model of *nts1116*+AON. This can be understood as the ratio of the number of nodules inhibited by the virtual experiment to the number of nodules inhibited by a real Bragg plant. The similarity degrees of overall nodule number produced by virtual experiments on the 10^th^ and the 16^th^ day after sowing are listed in Fig. 3 and Fig. 4, where *R_q_* and *R_sdi_* represent the transport rates of Q and SDI signals (mm/day). These data indicated that the similarity degrees resulting from cotyledon-shoot hypothesis were generally much higher than those from cotyledon-root hypothesis, supporting the former hypothesis. Considering that values of *S_cp_* greater than 100% may mean over-regulation and might not be optimal, the criterion for further evaluating *S_cp_* is defined in Fig. 5. According to this criterion, the virtual experiments based on cotyledon-root hypothesis produced unsatisfactory results on the 10^th^ day (Fig. 3, left-hand column), in sharp contrast to the cotyledon-shoot experiments (Fig. 3, right-hand column). Although there were good results derived from virtual experiments CRH_1, CRH_2, CRH_11 and CRH_13 on the 16^th^ day (Fig. 4, left-hand column) in terms of nodule number, the nodule size and density from these experiments were all far from similar with the Bragg pattern (Fig. 6). In comparison, the nodule distribution generated by CSH_1 (Fig. 6*D*) – the opposite of CRH_1 – was quite close to that of the Bragg architectural model.

We predicted from these complementation experiments that the cotyledons should be part of the shoot and participate as an SDI producer in wild-type soybean plants.

### Confirmation of the Virtual-Experiment Result

To confirm the above prediction and also to evaluate the effectiveness of this approach, a “real-plant” grafting experiment was conducted. The critical experiment was to graft – between Bragg and *nts1116* plants – the shoot of one genotype with cotyledons to the root of the other genotype without cotyledons, and also to graft the shoot of one genotype without cotyledons to the root of the other genotype with cotyledons, forming four graft combinations: Ns+Nc+Br, Ns+Bc+Br, Bs+Bc+Nr and Bs+Nc+Nr (Table 1). Materials and methods for this graft experiment are given in the supporting Text S1. The collected empirical data for nodule number were not only classified by each graft type but also according to each plant's cotyledon retention status (Table 2).

**Table 1:** Real-plant graft types.

Ns+Nc+Br	*nts1116* shoot with cotyledons+Bragg root without cotyledons
Ns+Bc+Br	*nts1116* shoot without cotyledons+Bragg root with cotyledons
Bs+Bc+Nr	Bragg shoot with cotyledons+*nts1116* root without cotyledons
Bs+Nc+Nr	Bragg shoot without cotyledons+*nts1116* root with cotyledons

**Table 2:** Cotyledon retention status.

0_C	both cotyledons have fallen
1_YC	the plant only has one yellow cotyledon
2_YC	both cotyledons of the plant have turned yellow
2_GC	both cotyledons of the plant are green

**Figure 7:**
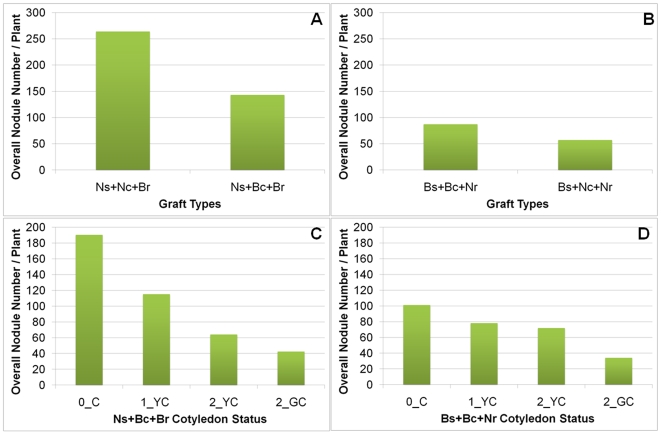
Nodulation of real-plant mutant-parent grafts. (A) Nodule numbers from Ns+Nc+Br (*nts1116* shoot with cotyledons + Bragg root without cotyledons) and Ns+Bc+Br (*nts1116* shoot without cotyledons + Bragg root with cotyledons) graft types. (B) Nodule numbers from Bs+Bc+Nr (Bragg shoot with cotyledons + *nts1116* root without cotyledons) and Bs+Nc+Nr (Bragg shoot without cotyledons + *nts1116* root with cotyledons) graft types. (C) Nodule numbers from Ns+Bc+Br plants classified by cotyledon retention status. (D) Nodule numbers from Bs+Bc+Nr plants classified by cotyledon retention status.

According to the experimental results, the nodule number from the Ns+Nc+Br graft type was much higher than that from the Ns+Bc+Br (Fig. 7*A*). For the Ns+Bc+Br graft type alone, its plants with fallen cotyledons had more nodules than those with persisting cotyledons, and the plants with yellow cotyledons had more nodules than those with green cotyledons (Fig. 7*C*). These differences suggest Bragg cotyledons were the only leaves to regulate nodulation in Ns+Bc+Br plants, because unifoliate and trifoliate leaves of *nts1116* plants were unable to do so.

Data of another graft type with Bragg cotyledons – the Bs+Bc+Nr (Fig. 7*D*) also suggested that the Bragg cotyledons participated in providing SDI. However, more nodules were found in the Bs+Bc+Nr plants than in the Bs+Nc+Nr plants that had no Bragg cotyledons (Fig. 7*B*). An explanation for this observation is that the Bs+Nc+Nr allowed more nodules to be formed at early stages than the Bs+Bc+Nr, leading to more Q signal moving from root to shoot. As the cotyledon biomass declined greatly at later stages of seedling growth (resources are unloaded for plant growth and the “spent” cotyledon is eventually discarded), the difference in shoot between Bs+Bc+Nr and Bs+Nc+Nr became insignificant. Therefore larger amounts of Q triggered more SDI, which finally inhibited more nodules in Bs+Nc+Nr.

**Figure 8:**
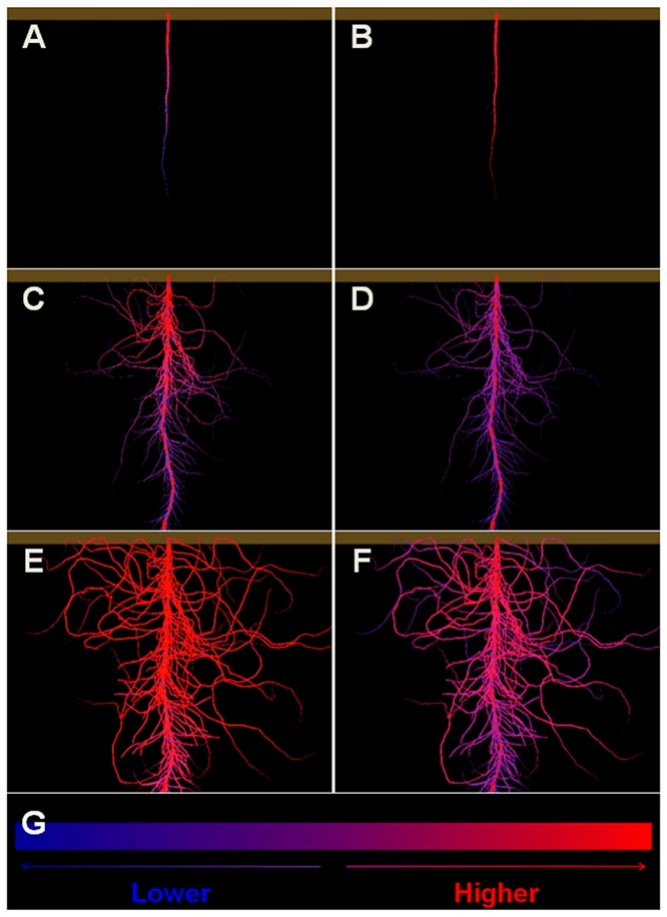
Allocation of the putative SDI signal during a virtual experiment. The allocation of SDI in root during CRH_1 was visualised on (A) the 5th, (C) the 10th, and (E) the 16th day post-sowing. Visualisations (B) (D) and (F) were from the functional-structural model used in CSH_1, respectively representing the 5th, 10th and 16th day. The colour scheme to represent signal concentration is given in (G).

To better understand this nonlinear characteristic brought out by real-plant experiments, we returned to the virtual-experiment models and visualised the dynamic signal allocation during CRH_1 and CSH_1 (Fig. 8). As demonstrated by the visualisation, the SDI concentration (in the root) of CRH_1 was lower than that of CSH_1 on the 5^th^ day but became higher from the 10^th^ day on, in agreement with the above analysis of the nodulation difference between Bs+Bc+Nr and Bs+Nc+Nr. Thus, we conclude that the testing result from our initial application of computational complementation is confirmed: the cotyledons “belong” to the shoot and function as a source of the nodulation regulator in wild-type soybeans.

## Discussion

The computational complementation approach introduced here is an original contribution to the study of legume autoregulation of nodulation. Compared with conventional biological technologies with broader implications to plant development, one of the major advantages of this approach is its capability to complement the deficiency of a mutant plant at an organ scale with totally hypothetical and concept-derived physiological components. It is also able to make hypothetical signalling details manipulable and visible. For example, as demonstrated in the above case, signal transport rates can be modified as hypothesised and the allocation of signal can be dynamically visualised. These functionalities not only enable AON researchers to test hypotheses or make predictions using time- and resource-saving virtual experiments, but also bring out possible underlying details that are unobservable through real-plant experiments. Moreover, the application of this approach is not only limited to AON research, but also potential to other plant signalling studies such as those on branching regulation (*e.g.,* [38]), flowering control (*e.g.*, [39]) and lateral root initiation (*e.g.,* [40]).

This approach contributes a new idea to the domain of computational plant modelling – computational complementation. From a classic modelling point of view, one can formulate a model based on empirical data and then verify the model against the data, which has been used for development of crop (*e.g.,* [41]) and architectural (*e.g.,* [42]) models. However, what we investigate is a largely unclear internal signalling system – most of the detailed mechanisms remain unknown, which determines there is no direct parameterisation-and-verification data to evaluate the modelled signalling hypotheses. Using an indirect strategy, functional-structural modelling allows us to use the observable structure as a reporter for estimation of the unobservable function. But for this study, we have to link the structure of one genotype with the function of another genotype. The reason for this is: the wild-type Bragg nodulation has already been regulated, thus incorporating AON to Bragg architecture would double the regulation and have no reasonable comparison target for validation; in contrast, the *nts1116* is a non-AON plant and this is its only difference with Bragg, therefore activating AON in *nts1116* plant could result in system behaviours comparable with the wild type.

Another feature of this approach resides in the level of complexity for simulation of structural and signalling processes. We captured root details for studying shoot-root signalling rather than oversimplifying the root system. And the signalling pathways are constructed with sub-modules of which the size and number can be manipulated without limitation, which allows future modelling work to be extended to lower-scale mechanisms (such as tissue and cellular scale). We also created a synchronisation algorithm for coordination of multi-rate procedures to enhance the precision of signalling-development interactions. A description of these modelling techniques is given in the supporting Text S2.

The approach also has some limitations. For example, due to the nature of complementation, it can only be used for a single mutation at a time, though leaky mutants can be handled by parameter optimization. Another drawback is that it cannot distinguish between different mutations in the same pathway that result in the same phenotype in the first instance. In other words, if the hypothesised mechanisms used to complement the mutant are the same in both cases, and so is the phenotype of the two mutants, computational complementation cannot be used to say which gene component of the regulatory network has been mutated.

Our first application of this approach was to test whether wild-type soybean cotyledons are involved in production of SDI. Also but more importantly, we expected this application to evaluate whether the computational complementation idea is effective. The virtual-experiment results suggested the wild-type cotyledons can produce SDI, which was further confirmed by a graft experiment on real plants. This demonstrates the feasibility of computational complementation and shows its usefulness for future applications.

The next step is to apply this approach to support research for the identification of Q and SDI. Candidate signals, such as CLE peptide for Q [13],[14] and auxin for SDI [43], will be tested to see if they play the roles in AON as hypothesised. In addition, environmental factors, such as soil nitrogen status, that have effects on the process could also be tested with this approach. Furthermore, the finding that wild-type soybean cotyledons act as an SDI producer in AON opens the door for testing physiological transgenerational effects, such as altered nodulation patterns influenced by the *Bradyrhizobium* infection status of mother plant through presence of SDI in cotyledons.

## Supporting Information

Text S1 Materials and methods for glasshouse experiments
(0.03 MB DOC)

Text S2 Growth data collection and model construction
(3.70 MB DOC)

Text S3 Assumptions and conditions for virtual experiments
(0.15 MB DOC)

Figure S1 Visualisation of nodule distribution with inhibited nodules on the 16th day post-sowing
(2.34 MB TIF)

## References

[1] Graham PH, Vance CP (2003). Legumes: importance and constraints to greater use.. Plant Physiol.

[2] Kinkema M, Scott PT, Gresshoff PM (2006). Legume nodulation: successful symbiosis through short- and long-distance signalling.. Funct Plant Biol.

[3] Oka-Kira E, Kawaguchi M (2006). Long-distance signaling to control root nodule number.. Curr Opin Plant Biol.

[4] Carroll BJ, McNeil DL, Gresshoff PM (1985). Isolation and properties of soybean [*Glycine max* (L.) Merr.] mutants that nodulate in the presence of high nitrate concentrations.. P Natl Acad Sci USA.

[5] Carroll BJ, McNeil DL, Gresshoff PM (1985). A supernodulation and nitrate-tolerant symbiotic (*nts*) soybean mutant.. Plant Physiol.

[6] Delves AC, Mathews A, Day DA, Carter AS, Carroll BJ (1986). Regulation of the soybean-rhizobium nodule symbiosis by shoot and root factors.. Plant Physiol.

[7] Gresshoff PM (2003). Post-genomic insights into plant nodulation symbioses.. Genome Biol.

[8] Nontachaiyapoom S, Scott PT, Men AE, Kinkema M, Schenk PM (2007). Promoters of orthologous *Glycine max* and *Lotus japonicus* nodulation autoregulation genes interchangeably drive phloem-specific expression in transgenic plants.. Mol Plant Microbe In.

[9] Searle IR, Men AE, Laniya TS, Buzas DM, Iturbe-Ormaetxe I (2003). Long-distance signaling in nodulation directed by a CLAVATA1-like receptor kinase.. Science.

[10] Miyahara A, Hirani TA, Oakes M, Kereszt A, Kobe B (2008). Soybean nodule autoregulation receptor kinase phosphorylates two kinase-associated protein phosphatases *in vitro*.. J Biol Chem.

[11] Krusell L, Madsen LH, Sato S, Aubert G, Genua A (2002). Shoot control of root development and nodulation is mediated by a receptor-like kinase.. Nature.

[12] Schnabel E, Journet E-P, Carvalho-Niebel Fd, Duc G, Frugoli J (2005). The *Medicago truncatula SUNN* gene encodes a *CLV1*-like leucine-rich repeat receptor kinase that regulates nodule number and root length.. Plant Mol Biol.

[13] Gresshoff PM, Lohar D, Chan P-K, Biswas B, Jiang Q (2009). Genetic analysis of ethylene regulation of legume nodulation.. Plant Signal Behav.

[14] Okamoto S, Ohnishi E, Sato S, Takahashi H, Nakazono M (2009). Nod factor/nitrate-induced CLE genes that drive HAR1-mediated systemic regulation of nodulation.. Plant Cell Physiol.

[15] Lin Y-H, Ferguson BJ, Kereszt A, Gresshoff PM (2010). Suppression of hypernodulation in soybean by a leaf-extracted, NARK- and Nod factor-dependent low molecular mass fraction.. New Phytol. In press.

[16] Kinkema M, Gresshoff PM (2008). Investigation of downstream signals of the soybean autoregulation of nodulation receptor kinase GmNARK.. Mol Plant Microbe In.

[17] Kitano H (2002). Systems biology: a brief overview.. Science.

[18] Minorsky PV (2003). Achieving the in silico plant. Systems biology and the future of plant biological research.. Plant Physiol.

[19] Hammer GL, Sinclair TR, Chapman SC, Oosterom Ev (2004). On systems thinking, systems biology, and the in silico plant.. Plant Physiol.

[20] Weng G, Bhalla US, Iyengar R (1999). Complexity in biological signaling systems.. Science.

[21] Neves SR, Iyengar R (2002). Modeling of signaling networks.. BioEssays.

[22] Jönsson H, Heisler MG, Shapiro BE, Meyerowitz EM, Mjolsness E (2006). An auxin-driven polarized transport model for phyllotaxis.. P Natl Acad Sci USA.

[23] Berleth T, Scarpella E, Prusinkiewicz P (2007). Towards the systems biology of auxin-transport-mediated patterning.. Trends Plant Sci.

[24] Rolland-Lagan A-G, Prusinkiewicz P (2005). Reviewing models of auxin canalization in the context of leaf vein pattern formation in Arabidopsis.. Plant J.

[25] Reuille PBd, Bohn-Courseau I, Ljung K, Morin H, Carraro N (2006). Computer simulations reveal properties of the cell-cell signaling network at the shoot apex in Arabidopsis.. P Natl Acad Sci USA.

[26] Godin C, Sinoquet H (2005). Functional–structural plant modelling.. New Phytol.

[27] Bidel LPR, Pagès L, Rivière LM, Pelloux G, Lorendeau JY (2000). MassFlowDyn I: a carbon transport and partitioning model for root system architecture.. Ann Bot-London.

[28] Allen M, Prusinkiewicz P, DeJong T (2005). Using L-systems for modeling source–sink interactions, architecture and physiology of growing trees: the L-PEACH model.. New Phytol.

[29] Drouet J-L, Pagès L (2007). GRAAL-CN: a model of GRowth, Architecture and ALlocation for Carbon and Nitrogen dynamics within whole plants formalised at the organ level.. Ecol Model.

[30] Janssen JM, Lindenmayer A (1987). Models for the control of branch positions and flowering sequences of capitula in *Mycelis muralis* (L.) dumont (compositae).. New Phytol.

[31] Prusinkiewicz P, Lindenmayer A (1990). The Algorithmic Beauty of Plants..

[32] Buck-Sorlin GH, Kniemeyer O, Kurth W (2005). Barley morphology, genetics and hormonal regulation of internode elongation modelled by a relational growth grammar.. New Phytol.

[33] Buck-Sorlin G, Hemmerling R, Kniemeyer O, Burema B, Kurth W (2008). A rule-based model of barley morphogenesis, with special respect to shading and gibberellic acid signal transduction.. Ann Bot-London.

[34] Prusinkiewicz P, Crawford S, Smith RS, Karin Ljung, Bennett T (2009). Control of Bud Activation by an Auxin Transport Switch.. P Natl Acad Sci USA. In press.

[35] Delves AC, Higgins A, Gresshoff P (1992). Shoot apex removal does not alter autoregulation of nodulation in soybean.. Plant Cell Environ.

[36] Kahl G (1995). Dictionary of Gene Technology..

[37] Hansen AP, Peoples MB, Gresshoff PM, Atkins CA, Pate JS (1989). Symbiotic performance of supernodulating soybean (*Glycine max* (L.) Merrill) mutants during development on different nitrogen regimes.. J Exp Bot.

[38] Dun EA, Hanan J, Beveridge CA (2009). Computational modeling and molecular physiology experiments reveal new insights into shoot branching in pea.. Plant Cell. In press.

[39] Wenden B, Dun EA, Hanan J, Andrieu B, Weller JL (2009). Computational analysis of flowering in pea (*Pisum sativum*).. New Phytol.

[40] Aloni R, AloniI E, Langhans M, Ullrich CI (2006). Role of cytokinin and auxin in shaping root architecture: regulating vascular differentiation, lateral root initiation, root apical dominance and root gravitropism.. Ann Bot-London.

[41] Jones JW, Keating BA, Porter CH (2001). Approaches to modular model development.. Agr Syst.

[42] Watanabe T, Hanan JS, Room PM, Hasegawa T, Nakagawa H (2005). Rice morphogenesis and plant Architecture: measurement, specification and the reconstruction of structural development by 3D architectural modelling.. Ann Bot-London.

[43] Mathesius U (2008). Auxin: at the root of nodule development?. Funct Plant Biol.

