# Supplementary material for: Computational Complementation: A Modelling Approach to Study Signalling Mechanisms during Legume Autoregulation of Nodulation
Source: PLoS Comput Biol. 2010 Feb 26;6(2):e1000685. doi: 10.1371/journal.pcbi.1000685 (PMC2829028; doi:10.1371/journal.pcbi.1000685)
Supplement: Text S1 — Materials and methods for glasshouse experiments (0.03 MB DOC) [file pcbi.1000685.s001.doc]

# Text S1: Materials and methods for glasshouse experiments

**Soybean growth experiment for architectural data collection**

Soybean seeds of wild type parent Bragg and derived mutant *nts1116* (surface-sterilised with 3% H_2_O_2_ in 70% ethanol for 3 minutes) were sown into autoclaved pots (20 cm in diameter) filled with autoclaved grade 2 vermiculite. The pots were then watered until complete saturation. All plants were kept in an air-conditioned glasshouse with controlled temperature of 28 ºC during day and 23 ºC at night. On the day after sowing, the seedlings were inoculated with a commercial peat containing *Bradyrhizobium japonicum* strain CB1809 (130 g peat CB1809 mixed with 2,800 mL water). Five plants were kept in each pot after culling on the fifth day. The plants were fertilised twice during this experiment – on the third day and the eighth day after sowing - with B&D nutrient solution [1] plus 2 mM KNO_3_. This level of nitrate stimulates plant growth and has minimal effect on nodulation in soybean [2]. The developmental data of shoot and root components, such as internode, cotyledon, unifoliate leaf, trifoliate leaf and leaflets, primary root, lateral root and nodules (including nodule number and nodule distribution) were collected every two days from the third day after sowing by destructive sampling of five plants for each genotype.

**Soybean graft experiment**

Soybean seeds of Bragg and *nts1116* were surface-sterilised, planted, and grown as described above. Grafting in the hypocotyl or epicotyl was carried out ten days post-sowing and the grafted plants were inoculated with a liquid YMB culture (2 g/L mannitol, 0.4 g/L yeast extract, 0.5 g/L K_2_HPO_4_, 0.2 g/L MgSO_4_·7H_2_O, 0.1 g/L NaCl, pH 6.8) of CB1809 on the day after grafting. The plants were irrigated with B&D nutrient plus 1 mM KNO_3_ solution. The nodule numbers were scored from the grafted plants two weeks after grafting.

**References**

1. Broughton WJ, Dilworth MJ (1971) Control of leghaemoglobin synthesis in snake beans. Biochem J 125: 1075-1080.

2. Carroll BJ, McNeil DL, Gresshoff PM (1985) Isolation and properties of soybean [*Glycine max* (L.) Merr.] mutants that nodulate in the presence of high nitrate concentrations. P Natl Acad Sci USA 82: 4162-4166.
