# Supplementary material for: Computational Complementation: A Modelling Approach to Study Signalling Mechanisms during Legume Autoregulation of Nodulation
Source: PLoS Comput Biol. 2010 Feb 26;6(2):e1000685. doi: 10.1371/journal.pcbi.1000685 (PMC2829028; doi:10.1371/journal.pcbi.1000685)
Supplement: Text S2 — Growth data collection and model construction (3.70 MB DOC) [file pcbi.1000685.s002.doc]

# Text S2: Growth data collection and model construction

**Methods for growth data collection**

To collect growth data, the shoot structure was mapped as sequences of symbols [1] where each symbol represents a particular type of plant component (such as internode, cotyledon, petiole and leaflet). For the root system, we classify its components as primary root, first-order lateral roots and nodules. The second-order lateral roots were not taken into account in this work. To capture the branching pattern and help in identifying the first-order lateral roots, we developed an “RULD” root mapping method [2] (Figure 1). We also developed a nodule positioning method to help in recording the distribution information of nodulation (Figure 2).


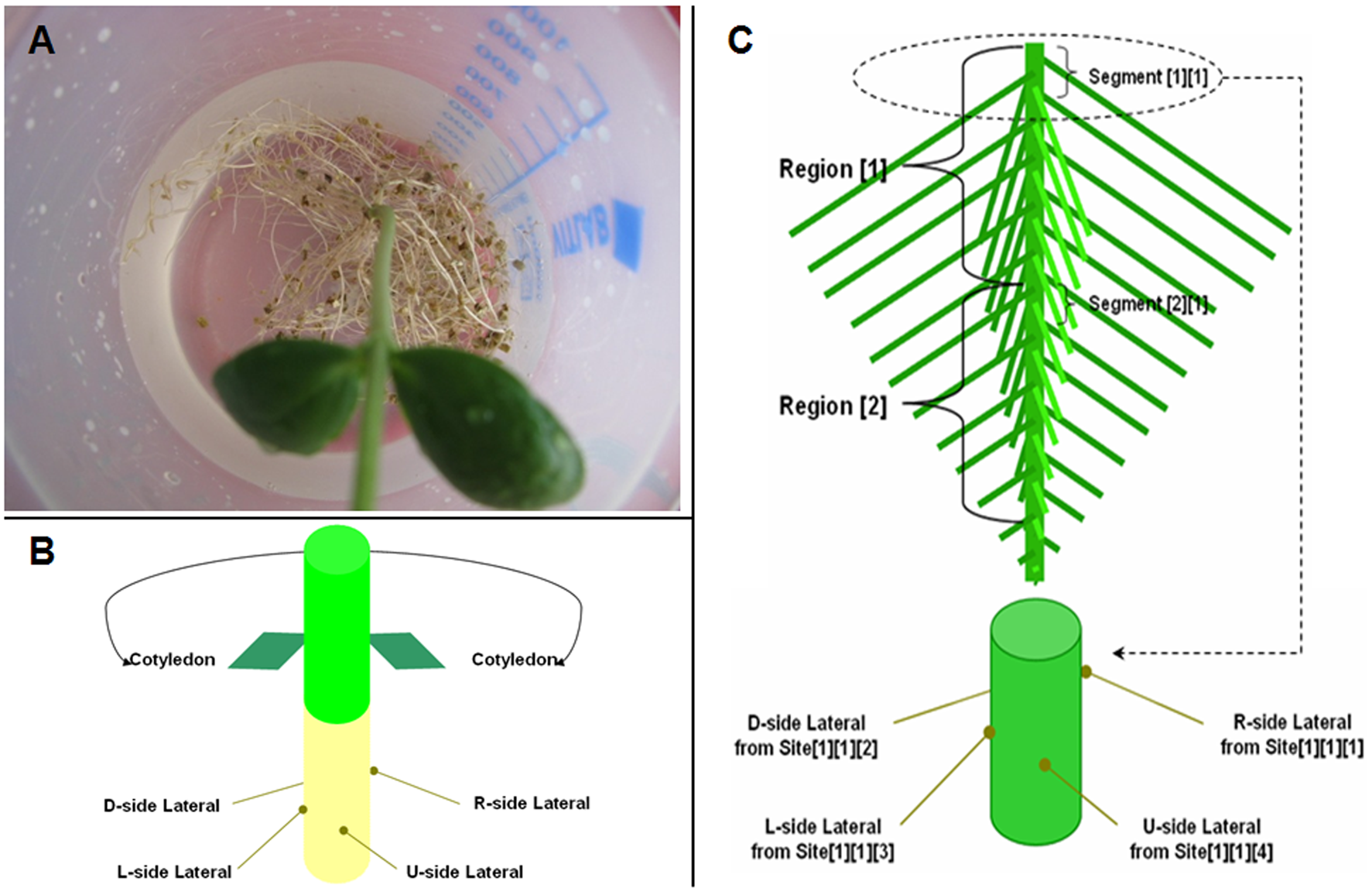


**Figure 1. The “RULD” root mapping method.** Based on the observed lateral root branching pattern from soybean plants (as shown in image A), the first-order lateral roots were characterised into four sides (R, U, L and D) according to the relative positions of their starting points to the obtuse angle composed by cotyledons in the horizontal plane (as demonstrated in image B). Then the first-order lateral roots were further classified by regions (50 mm long), segments and sites (as illustrated in image C).


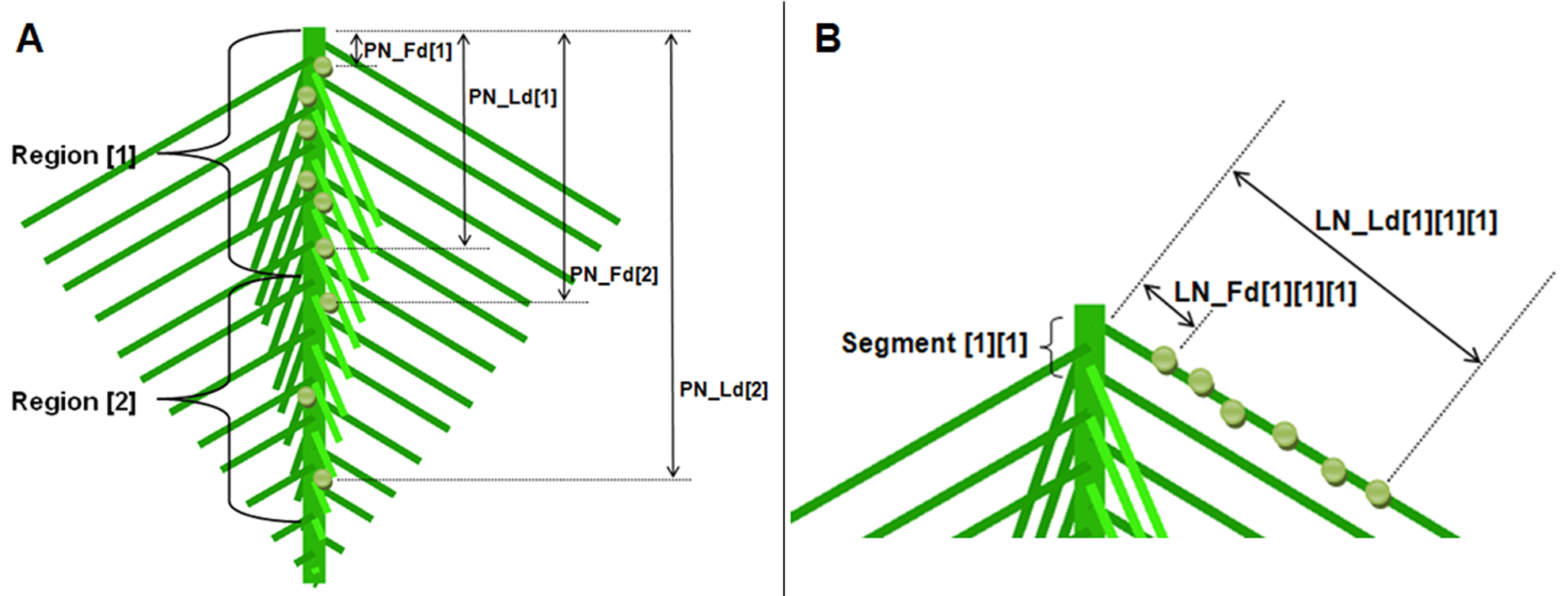


**Figure 2. Method for recording distribution information of nodulation.** (*A*) For each region on the primary root (as defined in Figure 1), the distance between the first nodule and the primary-root starting point was measured and recorded as “PN_Fd”; the distance between the last nodule and the primary-root starting point was measured and recorded as “PN_Ld”. (*B*) For the lateral root nodules, the same method was used with the distances (LN_Fd and LN_Ld) measured from the lateral-root starting point. The positions of the first nodule and the last nodule determine the location of a nodulation section. The nodule density for a section was calculated based on its length and the number of nodules within the section.

**Growth data**

In analysing the raw data collected with the above methods, some mean values decreased with increasing day, due to the destructive sampling for measurement. The data (except for cotyledon dry weight) were processed to remove this anomaly by not allowing values to decrease, so that normal growth patterns could be simulated. For example, if and represent the length of an internode on the 6^th^ day and the 8^th^ day and if is bigger than , then the value of is changed to be the same as . The processed architectural data are given in Tables 1 - 12. The data on *nts1116* leaf dry weight, needed for building the functional-structural model, are given in Table 13.

**Table 1.** Dimension of shoot organs (Bragg).

| **Day**  **Organ Dimension (mm)** | | | | | **4** | **6** | **8** | **10** | **12** | **14** | **16** |
| --- | --- | --- | --- | --- | --- | --- | --- | --- | --- | --- | --- |
| Phytomer 1 | Internode | Length | | | 17.3 | 33.0 | 65.6 | 77.4 | 77.4 | 77.4 | 77.4 |
|  |  | Diameter | | | 2.1 | 2.3 | 2.6 | 2.9 | 2.9 | 2.9 | 2.9 |
|  | Cotyledon 1 | Length | | | 16.6 | 20.1 | 29.6 | 30.9 | 31.2 | 31.2 | 31.2 |
|  |  | Width | | | 8.8 | 12.2 | 17.2 | 18.6 | 18.6 | 18.6 | 18.6 |
|  | Cotyledon 2 | Length | | | 16.6 | 20.1 | 29.8 | 29.9 | 30.6 | 30.6 | 30.6 |
|  |  | Width | | | 8.8 | 12.2 | 16.8 | 16.8 | 18.0 | 18.0 | 18.0 |
| Phytomer 2 | Internode | Length | | | 0 | 0 | 20.7 | 54.7 | 69.2 | 85.8 | 95.2 |
|  |  | Diameter | | | 0 | 0 | 1.8 | 1.9 | 2.0 | 2.1 | 2.4 |
|  | Unifoliate 1 | Petiole | Length | | 0 | 0.8 | 5.2 | 9.8 | 10.6 | 11.8 | 15.5 |
|  |  |  | Diameter | | 0 | 0 | 1.1 | 1.1 | 1.1 | 1.3 | 1.3 |
|  |  | Surface | Length | | 0 | 10.5 | 12.0 | 18.9 | 23.2 | 26.0 | 27.0 |
|  |  |  | Width | | 0 | 4.2 | 8.2 | 19.0 | 26.0 | 30.6 | 34.8 |
|  | Unifoliate 2 | Petiole | Length | | 0 | 0.8 | 5.1 | 8.2 | 12.2 | 15.2 | 18.8 |
|  |  |  | Diameter | | 0 | 0 | 1.1 | 1.1 | 1.1 | 1.2 | 1.5 |
|  |  | Surface | Length | | 0 | 10.5 | 10.5 | 19.0 | 27.6 | 31.2 | 33.5 |
|  |  |  | Width | | 0 | 4.2 | 6.3 | 18.8 | 31.2 | 37.2 | 44.6 |
| Phytomer 3 | Internode | Length | | | 0 | 0 | 0 | 0 | 9.6 | 32.8 | 52.0 |
|  |  | Diameter | | | 0 | 0 | 0 | 0 | 1.7 | 2.2 | 2.7 |
|  | Trifoliate | Petiole | Length | | 0 | 0 | 0 | 0 | 7.0 | 26.6 | 49.2 |
|  |  |  | Diameter | | 0 | 0 | 0 | 0 | 1.3 | 2.8 | 2.8 |
|  |  | Leaflet 1 | Petiolule | Length | 0 | 0 | 0 | 0 | 3.0 | 4.4 | 5.7 |
|  |  |  |  | Diameter | 0 | 0 | 0 | 0 | 0.9 | 1.2 | 1.5 |
|  |  |  | Surface | Length | 0 | 0 | 0 | 10.4 | 25.8 | 59.2 | 80.2 |
|  |  |  |  | Width | 0 | 0 | 0 | 6.0 | 14.8 | 36.0 | 49.2 |
|  |  | Leaflet 2 | Petiolule | Length | 0 | 0 | 0 | 0 | 1.8 | 2.0 | 3.1 |
|  |  |  |  | Diameter | 0 | 0 | 0 | 0 | 0.8 | 1.0 | 1.4 |
|  |  |  | Surface | Length | 0 | 0 | 0 | 0 | 23.4 | 51.2 | 72.7 |
|  |  |  |  | Width | 0 | 0 | 0 | 0 | 9.2 | 30.0 | 39.3 |
|  |  | Leaflet 3 | Petiolule | Length | 0 | 0 | 0 | 0 | 1.8 | 2.0 | 3.1 |
|  |  |  |  | Diameter | 0 | 0 | 0 | 0 | 0.8 | 1.0 | 1.3 |
|  |  |  | Surface | Length | 0 | 0 | 0 | 0 | 22.0 | 51.8 | 75.1 |
|  |  |  |  | Width | 0 | 0 | 0 | 0 | 9.0 | 34.6 | 40.5 |
| Phytomer 4 | Internode | Length | | | 0 | 0 | 0 | 0 | 0 | 3.4 | 13.2 |
|  |  | Diameter | | | 0 | 0 | 0 | 0 | 0 | 1.3 | 2.1 |
|  | Trifoliate | Petiole | | Length | 0 | 0 | 0 | 0 | 0 | 2.8 | 15.5 |
|  |  |  |  | Diameter | 0 | 0 | 0 | 0 | 0 | 1.0 | 1.5 |
|  |  | Leaflet 1 | Petiolule | Length | 0 | 0 | 0 | 0 | 0 | 2.2 | 5.7 |
|  |  |  |  | Diameter | 0 | 0 | 0 | 0 | 0 | 0.8 | 1.3 |
|  |  |  | Surface | Length | 0 | 0 | 0 | 0 | 0 | 19.0 | 46.6 |
|  |  |  |  | Width | 0 | 0 | 0 | 0 | 0 | 8.8 | 27.6 |
|  |  | Leaflet 2 | Petiolule | Length | 0 | 0 | 0 | 0 | 0 | 1.0 | 3.0 |
|  |  |  |  | Diameter | 0 | 0 | 0 | 0 | 0 | 0.7 | 1.2 |
|  |  |  | Surface | Length | 0 | 0 | 0 | 0 | 0 | 15.6 | 34.5 |
|  |  |  |  | Width | 0 | 0 | 0 | 0 | 0 | 4.6 | 20.2 |
|  |  | Leaflet 3 | Petiolule | Length | 0 | 0 | 0 | 0 | 0 | 1.0 | 2.7 |
|  |  |  |  | Diameter | 0 | 0 | 0 | 0 | 0 | 0.7 | 1.2 |
|  |  |  | Surface | Length | 0 | 0 | 0 | 0 | 0 | 16.6 | 33.9 |
|  |  |  |  | Width | 0 | 0 | 0 | 0 | 0 | 4.4 | 20.2 |

**Table 2.** Length of primary and lateral roots (Bragg).

| **Day**  **Root Length (mm)** | | **4** | **6** | **8** | **10** | **12** | **14** | **16** |
| --- | --- | --- | --- | --- | --- | --- | --- | --- |
| Primary root length | | 88.3 | 141.2 | 231.4 | 293.2 | 301.7 | 301.7 | 303.8 |
| Average lateral root length | Region 1 | 0 | 10 | 73.6 | 135.9 | 135.9 | 135.9 | 151.7 |
|  | Region 2 | 0 | 0 | 18.4 | 32.5 | 47.2 | 53.4 | 53.4 |
|  | Region 3 | 0 | 0 | 7 | 18.4 | 37.9 | 37.9 | 37.9 |
|  | Region 4 | 0 | 0 | 0 | 16.3 | 27.3 | 27.3 | 34 |
|  | Region 5 | 0 | 0 | 0 | 0 | 8 | 20.3 | 24 |

**Table 3.** Number of primary and lateral root nodules (Bragg).

| **Day**  **Nodule Number** | | **4** | **6** | **8** | **10** | **12** | **14** | **16** |
| --- | --- | --- | --- | --- | --- | --- | --- | --- |
| Region 1 | Number of primary root nodules | 0 | 0 | 2 | 2 | 5 | 5 | 5 |
|  | Number of lateral root nodules (overall) | 0 | 0 | 0 | 0 | 2 | 19 | 25 |
| Region 2 | Number of primary root nodules | 0 | 0 | 0 | 0 | 2 | 2 | 2 |
|  | Number of lateral root nodules (overall) | 0 | 0 | 0 | 0 | 0 | 2 | 14 |
| Region 3 | Number of primary root nodules | 0 | 0 | 0 | 0 | 1 | 1 | 1 |
|  | Number of lateral root nodules (overall) | 0 | 0 | 0 | 0 | 0 | 0 | 2 |
| Region 4 | Number of primary root nodules | 0 | 0 | 0 | 0 | 0 | 0 | 0 |
|  | Number of lateral root nodules (overall) | 0 | 0 | 0 | 0 | 0 | 0 | 0 |
| Region 5 | Number of primary root nodules | 0 | 0 | 0 | 0 | 0 | 0 | 0 |
|  | Number of lateral root nodules (overall) | 0 | 0 | 0 | 0 | 0 | 0 | 0 |

**Table 4.** Data for nodule distribution (Bragg).

| **Region** | **Root Type** | **Position of First Nodule (mm)** | **Position of Last Nodule (mm)** | **Nodule Interval (mm)** |
| --- | --- | --- | --- | --- |
| Region 1 | Primary root | 8.5 | 25.4 | 6.1 |
|  | Lateral roots (average) | 29.5 | 87.3 | 16.5 |
| Region 2 | Primary root | 67.3 | 77.1 | 12.6 |
|  | Lateral roots (average) | 44.8 | 52.3 | 3.8 |
| Region 3 | Primary root | 114.5 | 121.1 | 10.6 |
|  | Lateral roots (average) | N/A | N/A | N/A |
| Region 4 | Primary root | N/A | N/A | N/A |
|  | Lateral roots (average) | N/A | N/A | N/A |
| Region 5 | Primary root | N/A | N/A | N/A |
|  | Lateral roots (average) | N/A | N/A | N/A |

**Table 5.** Data for nodule growth (Bragg).

| **Days from nodule initialisation to appearance** | **Nodule diameter expansion rate (mm/day)** | **Maximum nodule diameter (mm)** |
| --- | --- | --- |
| 2 | 0.19 | 2.6 |

**Table 6.** Data for lateral root distribution (Bragg).

| **Region** | **Number of Segments** | **Lateral Interval (mm)** | **Probability of Lateral Root Generation** | | | |
| --- | --- | --- | --- | --- | --- | --- |
|  |  |  | **R Laterals** | **U Laterals** | **L Laterals** | **D Laterals** |
| Region 1 | 17 | 0.7 | 0.7 | 0.7 | 0.5 | 0.6 |
| Region 2 | 16 | 0.8 | 0.6 | 0.6 | 0.6 | 0.6 |
| Region 3 | 14 | 0.9 | 0.6 | 0.5 | 0.5 | 0.4 |
| Region 4 | 13 | 1 | 0.4 | 0.3 | 0.4 | 0.5 |
| Region 5 | 12 | 1 | 0.4 | 0.4 | 0.5 | 0.2 |

**Table 7.** Dimension of shoot organs (*nts1116*).

| **Day**  **Organ Dimension (mm)** | | | | | **4** | **6** | **8** | **10** | **12** | **14** | **16** |
| --- | --- | --- | --- | --- | --- | --- | --- | --- | --- | --- | --- |
| Phytomer 1 | Internode | Length | | | 20.4 | 42.2 | 77.8 | 77.8 | 77.8 | 77.8 | 79.6 |
|  |  | Diameter | | | 1.7 | 1.7 | 2.3 | 2.3 | 2.3 | 2.4 | 2.4 |
|  | Cotyledon 1 | Length | | | 13.6 | 20.6 | 25.6 | 26.8 | 26.8 | 26.8 | 26.8 |
|  |  | Width | | | 8.8 | 12.2 | 15.4 | 16 | 16 | 16.3 | 16.3 |
|  | Cotyledon 2 | Length | | | 13.6 | 20.2 | 25.4 | 26.2 | 26.2 | 26.2 | 26.8 |
|  |  | Width | | | 8.8 | 12.6 | 15.8 | 16.2 | 16.2 | 16.2 | 16.2 |
| Phytomer 2 | Internode | Length | | | 0 | 0 | 21.6 | 54.4 | 80.9 | 83.3 | 83.3 |
|  |  | Diameter | | | 0 | 0 | 1.4 | 1.6 | 1.8 | 2 | 2.1 |
|  | Unifoliate 1 | Petiole | Length | | 0 | 1.8 | 3.6 | 7 | 13 | 14.3 | 14.3 |
|  |  |  | Diameter | | 0 | 0 | 0.7 | 0.9 | 1.1 | 1.2 | 1.2 |
|  |  | Surface | Length | | 0 | 11 | 15.2 | 26.6 | 30.9 | 31 | 37.6 |
|  |  |  | Width | | 0 | 4.4 | 12.4 | 25 | 36.6 | 38 | 41.2 |
|  | Unifoliate 2 | Petiole | Length | | 0 | 1.8 | 3.8 | 6.8 | 11.9 | 19.5 | 19.5 |
|  |  |  | Diameter | | 0 | 0 | 0.8 | 0.9 | 1.3 | 1.4 | 1.4 |
|  |  | Surface | Length | | 0 | 11 | 17.6 | 24.8 | 32.4 | 37.3 | 38.4 |
|  |  |  | Width | | 0 | 4.4 | 15 | 24.6 | 37.8 | 38.5 | 44.4 |
| Phytomer 3 | Internode | Length | | | 0 | 0 | 0 | 0 | 7 | 21.7 | 28.6 |
|  |  | Diameter | | | 0 | 0 | 0 | 0 | 1.4 | 1.8 | 2.1 |
|  | Trifoliate | Petiole | Length | | 0 | 0 | 0 | 0 | 3.9 | 16.8 | 23.4 |
|  |  |  | Diameter | | 0 | 0 | 0 | 0 | 1.1 | 1.3 | 1.6 |
|  |  | Leaflet 1 | Petiolule | Length | 0 | 0 | 0 | 0 | 2 | 5 | 6.2 |
|  |  |  |  | Diameter | 0 | 0 | 0 | 0 | 0.6 | 1.2 | 1.3 |
|  |  |  | Surface | Length | 0 | 0 | 0 | 8.2 | 16.8 | 42.5 | 55.4 |
|  |  |  |  | Width | 0 | 0 | 0 | 2.4 | 4.9 | 26 | 34.4 |
|  |  | Leaflet 2 | Petiolule | Length | 0 | 0 | 0 | 0 | 1.6 | 2.3 | 2.3 |
|  |  |  |  | Diameter | 0 | 0 | 0 | 0 | 0.6 | 1 | 1.1 |
|  |  |  | Surface | Length | 0 | 0 | 0 | 0 | 14.5 | 37.2 | 50.6 |
|  |  |  |  | Width | 0 | 0 | 0 | 0 | 4 | 27 | 28.4 |
|  |  | Leaflet 3 | Petiolule | Length | 0 | 0 | 0 | 0 | 1.5 | 2.5 | 2.5 |
|  |  |  |  | Diameter | 0 | 0 | 0 | 0 | 0.6 | 1 | 1.1 |
|  |  |  | Surface | Length | 0 | 0 | 0 | 0 | 14.3 | 40.7 | 51.4 |
|  |  |  |  | Width | 0 | 0 | 0 | 0 | 3.9 | 20.7 | 29 |
| Phytomer 4 | Internode | Length | | | 0 | 0 | 0 | 0 | 0 | 3 | 3.6 |
|  |  | Diameter | | | 0 | 0 | 0 | 0 | 0 | 1.4 | 1.4 |
|  | Trifoliate | Petiole | | Length | 0 | 0 | 0 | 0 | 0 | 2.3 | 3.6 |
|  |  |  |  | Diameter | 0 | 0 | 0 | 0 | 0 | 0.8 | 1 |
|  |  | Leaflet 1 | Petiolule | Length | 0 | 0 | 0 | 0 | 0 | 1.5 | 2.8 |
|  |  |  |  | Diameter | 0 | 0 | 0 | 0 | 0 | 0.8 | 0.9 |
|  |  |  | Surface | Length | 0 | 0 | 0 | 0 | 0 | 14.8 | 20.8 |
|  |  |  |  | Width | 0 | 0 | 0 | 0 | 0 | 4 | 10.4 |
|  |  | Leaflet 2 | Petiolule | Length | 0 | 0 | 0 | 0 | 0 | 0.8 | 1.4 |
|  |  |  |  | Diameter | 0 | 0 | 0 | 0 | 0 | 0.7 | 0.8 |
|  |  |  | Surface | Length | 0 | 0 | 0 | 0 | 0 | 11.8 | 16 |
|  |  |  |  | Width | 0 | 0 | 0 | 0 | 0 | 3 | 7.2 |
|  |  | Leaflet 3 | Petiolule | Length | 0 | 0 | 0 | 0 | 0 | 1 | 1.4 |
|  |  |  |  | Diameter | 0 | 0 | 0 | 0 | 0 | 0.6 | 0.8 |
|  |  |  | Surface | Length | 0 | 0 | 0 | 0 | 0 | 11.8 | 17.2 |
|  |  |  |  | Width | 0 | 0 | 0 | 0 | 0 | 3.2 | 7 |

**Table 8.** Length of primary and lateral roots (*nts1116*).

| **Day**  **Root Length (mm)** | | **4** | **6** | **8** | **10** | **12** | **14** | **16** |
| --- | --- | --- | --- | --- | --- | --- | --- | --- |
| Primary root length | | 80.8 | 164.4 | 214.8 | 231.5 | 243 | 243 | 253.8 |
| Average lateral root length | Region 1 | 0 | 12.9 | 65.1 | 98.2 | 98.2 | 138.4 | 141.4 |
|  | Region 2 | 0 | 0 | 17.4 | 24.3 | 33.5 | 33.5 | 33.5 |
|  | Region 3 | 0 | 0 | 5.4 | 14.4 | 16.1 | 16.4 | 28.5 |
|  | Region 4 | 0 | 0 | 0 | 10 | 32.3 | 32.3 | 32.3 |
|  | Region 5 | 0 | 0 | 0 | 2.8 | 13.1 | 13.1 | 20.8 |

**Table 9.** Number of primary and lateral root nodules (*nts1116*).

| **Day**  **Nodule Number** | | **4** | **6** | **8** | **10** | **12** | **14** | **16** |
| --- | --- | --- | --- | --- | --- | --- | --- | --- |
| Region 1 | Number of primary root nodules | 0 | 0 | 7 | 14 | 14 | 14 | 14 |
|  | Number of lateral root nodules (overall) | 0 | 0 | 0 | 5 | 53 | 74 | 94 |
| Region 2 | Number of primary root nodules | 0 | 0 | 6 | 6 | 8 | 8 | 8 |
|  | Number of lateral root nodules (overall) | 0 | 0 | 0 | 0 | 2 | 9 | 13 |
| Region 3 | Number of primary root nodules | 0 | 0 | 0 | 1 | 3 | 5 | 5 |
|  | Number of lateral root nodules (overall) | 0 | 0 | 0 | 0 | 0 | 7 | 7 |
| Region 4 | Number of primary root nodules | 0 | 0 | 0 | 0 | 0 | 0 | 0 |
|  | Number of lateral root nodules (overall) | 0 | 0 | 0 | 0 | 0 | 0 | 0 |
| Region 5 | Number of primary root nodules | 0 | 0 | 0 | 0 | 0 | 0 | 0 |
|  | Number of lateral root nodules (overall) | 0 | 0 | 0 | 0 | 0 | 0 | 0 |

**Table 10.** Data for nodule distribution (*nts1116*).

| **Region** | **Root Type** | **Position of First Nodule (mm)** | **Position of Last Nodule (mm)** | **Nodule Interval (mm)** |
| --- | --- | --- | --- | --- |
| Region 1 | Primary root | 6.3 | 43.9 | 2.5 |
|  | Lateral roots (average) | 11.2 | 82.6 | 6.5 |
| Region 2 | Primary root | 55.5 | 88.9 | 4.5 |
|  | Lateral roots (average) | 15.5 | 31.8 | 3.3 |
| Region 3 | Primary root | 107.8 | 124 | 7.4 |
|  | Lateral roots (average) | 11.5 | 18.5 | 3.5 |
| Region 4 | Primary root | N/A | N/A | N/A |
|  | Lateral roots (average) | N/A | N/A | N/A |
| Region 5 | Primary root | N/A | N/A | N/A |
|  | Lateral roots (average) | N/A | N/A | N/A |

**Table 11.** Data for nodule growth (*nts1116*).

| **Days from nodule initialisation to appearance** | **Nodule diameter expansion rate (mm/day)** | **Maximum nodule diameter (mm)** |
| --- | --- | --- |
| 2 | 0.16 | 2.6 |

**Table 12.** Data for lateral root distribution (*nts1116*).

| **Region** | **Number of Segments** | **Lateral Interval (mm)** | **Probability of Lateral Root Generation** | | | |
| --- | --- | --- | --- | --- | --- | --- |
|  |  |  | **R Laterals** | **U Laterals** | **L Laterals** | **D Laterals** |
| Region 1 | 16 | 0.8 | 0.6 | 0.6 | 0.6 | 0.7 |
| Region 2 | 13 | 0.9 | 0.6 | 0.5 | 0.6 | 0.5 |
| Region 3 | 12 | 1.1 | 0.5 | 0.6 | 0.5 | 0.4 |
| Region 4 | 13 | 1 | 0.4 | 0.3 | 0.4 | 0.4 |
| Region 5 | 6 | 2.1 | 0.4 | 0.5 | 0.6 | 0.3 |

**Table 13.** Leaf dry weight (*nts1116*).

| **Day**  **Leaf** | | **4** | **6** | **8** | **10** | **12** | **14** | **16** |
| --- | --- | --- | --- | --- | --- | --- | --- | --- |
| Phytomer 1 | Cotyledon 1 | 63.9 mg | 46.8 mg | 41.4 mg | 33.9 mg | 25.8 mg | 24.9 mg | 22.8 mg |
|  | Cotyledon 2 | 63.9 mg | 46.8 mg | 41.4 mg | 33.9 mg | 25.8 mg | 24.9 mg | 22.8 mg |
| Phytomer 2 | Unifoliate 1 | 0 mg | 2.8 mg | 7.0 mg | 12.4 mg | 17.6 mg | 17.6 mg | 17.6 mg |
|  | Unifoliate 2 | 0 mg | 2.8 mg | 7.0 mg | 12.4 mg | 17.6 mg | 17.6 mg | 17.6 mg |
| Phytomer 3 | Trifoliate | 0 mg | 0 mg | 0 mg | 7.0 mg | 23.3 mg | 39.7 mg | 60.9 mg |
| Phytomer 4 | Trifoliate | 0 mg | 0 mg | 0 mg | 0 mg | 5.6 mg | 11.2 mg | 27.9 mg |

**Methods for model construction**

The architectural and functional-structural models were built with the L-system-based language “*cpfg*” in L-studio [3, 4]. In L-system-based architectural models [1, 5, 6], an organ-scale component (such as in internode) is usually simulated with a single module that not only contains its growth information but also represents its structure graphically. In this study, to enable the synchronisation of signalling and developmental processes with different rates and to visualise signal distribution details, we eliminate the graphical role from such modules and only used them to produce a set of standard “sub-modules” for simulation of shoot and root elongation. All sub-modules have the same length, defined as “UNIT” in this work, for both shoot and root structures. For example, for an internode, a leading module “I” is defined to restrict growth (such as elongation, etc.) based on empirical developmental data, while a set of sub-modules “D” play the role of making up structure of the internode. The addition of “D” sub-modules is determined by the empirical elongation data for this internode through the “I” module (Figure 3).


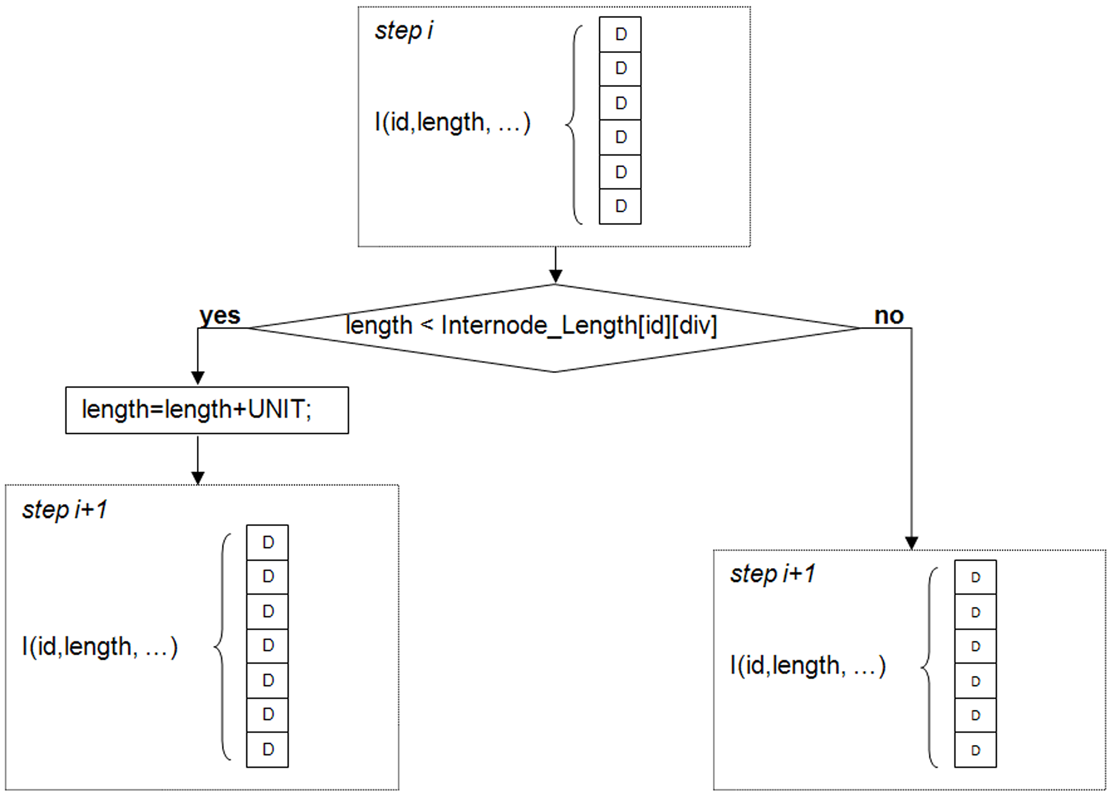


**Figure 3. Algorithm with sub-modules for internode elongation.** The leading module I(id,length,…) carries the basic developmental information of an internode, where “id” is the identification number of this internode and “length” is the current length of this internode. The array “Internode_Length[id][div]” contains the length information of all internodes over time, which is derived from empirical growth data. The “div” is the most basic time unit that can be defined by the user (e.g. it could be an hour or could also be a day). A “div” contains multiple L-system time steps. At a certain step i, if the length of an internode has not reached the maximum length for the current div (say, the value of Internode_Length[id][div] that corresponds to this internode), it will be increased with a standard length “UNIT”, meanwhile a sub-module “D” representing such an increment will be added to existing sub-modules to elongate the internode. Otherwise no addition will be made to the group of sub-modules and the internode length will remain at its previous value. The expressions in this flow chart are in C syntax.

The production of organ-scale components of the shoot is based on a set of developmental rules and relevant empirical data (Figure 4). These production rules are similar to those used in previous L-system-based architectural models [1, 5, 6].


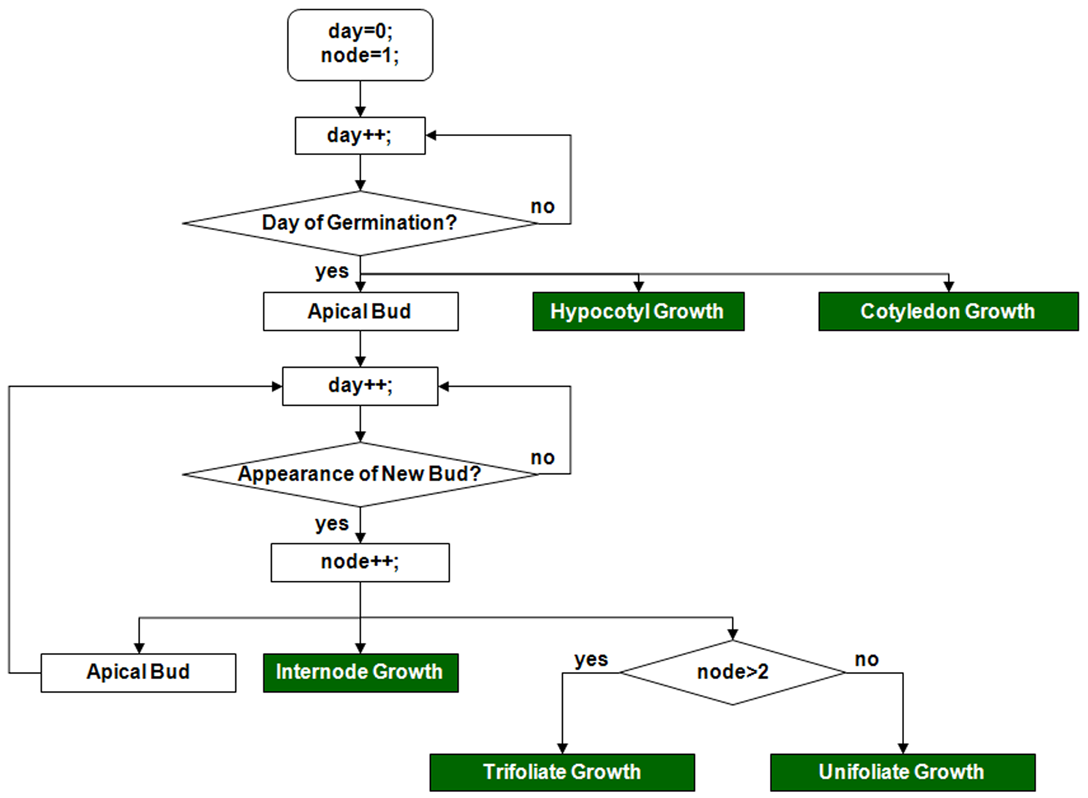


**Figure 4. Basic algorithm for production of shoot components.** The growth of main stem is led by an apical bud. Once the day of new bud appearance is met, a new node will be added and relevant organ components such as internode and leaf will be produced. The growth of these components (coloured in dark green) behaves individually and simultaneously with the advancement of the main stem. The expressions in this flow chart are in C syntax.

Compared with the clearly “ordered” shoot composition and growth, the development of root system is more complex. To model the elongation of the primary root, we use a module “K” to represent the root tip and to carry the developmental information. When the root tip is heading downwards, a set of sub-modules (with standard length UNIT) is added behind the “K” module to create the primary root structure. Meanwhile, the potential nodulation positions and lateral formation sites are also made available (Figure 5) according to empirical data collected with the root mapping (Figure 1) and nodule recording (Figure 2) methods.


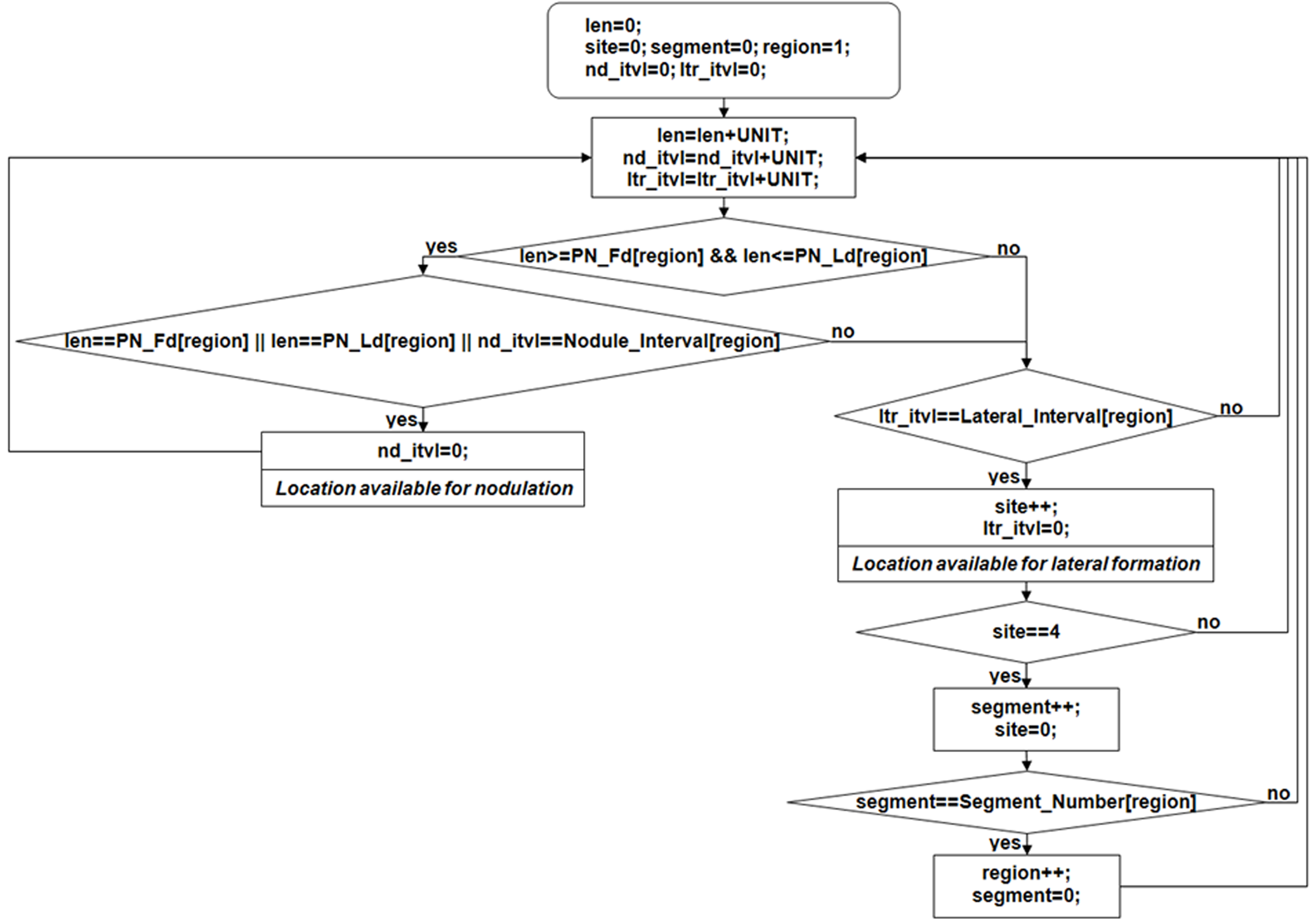


**Figure 5. Algorithm for primary root elongation.** The variable “len” represents the current length of primary root. The “site”, “segment” and “region” are counters to mark the site, segment and region (Figure 1) where the root tip is currently located. The “nd_itvl” and the “ltr_itvl” are counters to match the interval between two nodules and the interval between two sites. The values of these intervals vary depending on different regions. When the root length reaches the values of PN_Fd[region] or PN_Ld[region], or when it is between PN_Fd[region] and PN_Ld[region] (as shown by Figure 2) and the value of “nd_itvl” reaches the interval between two neighbouring nodules (represented by Nodule_Interval[region]), a location potential for nodule formation will be made available. When the root elongation covers a lateral site interval (represented by Lateral_Interval[region]), a site potential for lateral formation will be made available. When the site number reaches four, a new segment will be started. And when all segments in the current region (represented by Segment_Number[region]) are paved, a new region will be started. The expressions in this flow chart are in C syntax.

In the functional-structural model where the signalling activities are enabled, the signal transport between two neighbouring sub-modules is simulated with context-sensitive L-systems [4]. Compared with traditional context-sensitive L-system modelling where signals are passed from one organ (such as an internode) directly to another, the use of sub-modules (with standard length UNIT) here allows the signals to flow through and/or stop at a certain point within an organ. This improves the accuracy of signal transport, from a spatial point of view. And it also enables the visualisation of signal concentration variation at different locations of the same organ. However, since the developmental events (such as elongation) and the signalling events (such as signal transport) are based on various rates, the capability of signal exchange between two sub-modules was not enough and a coordination mechanism was required to synchronise these multi-rate events. We therefore developed a multi-rate synchronisation algorithm for the functional-structural model (Figure 6). The key role of this algorithm is still played by the standard increment UNIT. In the functional-structural model, the UNIT is not only added to the growing structure as a spatial unit for elongation, but also used with L-system time steps as the basis of system clock (each single step corresponds with a single UNIT). A user-defined constant “DIV” is used in the algorithm to divide a whole day’s time into lower-scale time sections (e.g. 24 hours). Other constants and variables involved in the algorithm can be described as $(E,R,T,C,\tau,\lambda,\theta,u,d,a,step,div,day,counter)$ by Equations 1 - 11.

(1)

$E$ is a set of signalling and developmental events (such as signal transport, root elongation, internode elongation and so forth).

(2)

Each element in $E$ has two alternative values: 1 and 0. When is equal to 1, its corresponding event (event *i*) is in an “activated” mode and keep going; otherwise the corresponding event is in a “stopped” mode and keep waiting.

(3)

Elements in $R$ represent the transport or growth rates (mm/day) of elements in $E$.

$\tau:E\to R$ (4)

The $\tau$ means that a one-to-one mapping relationship exists between elements in $E$ and elements in $R$.

(5)

$u=UNIT$ (6)

$d=DIV$ (7)

(8)

Each element in $T$ is the number of time steps that it takes to extend or move through a distance equal to the value of in $R$; $u$is a constant equal to the value of UNIT; $d$ is a constant equal to the value of DIV; the relationship between $R$ and $T$ is represented by $\lambda$.

(9)

The value of $a$ is equal to the maximum value in $T$.

(10)

The set $C$ defines the number of available time steps remaining for advancement of each signalling or developmental event during a time section (divided by DIV from a day’s time).

$\theta:E\to C$ (11)

The definition of $\theta$ means that a one-to-one mapping relationship exists between elements in $E$ and elements in $C$.

Variable $step$ is used to count the number of L-system time steps conducted. From the start of system running, every group of $a$ time steps conducted by the system is counted by a variable $div$. Variable $day$ marks the current day. Variable $counter$ counts how many L-system time steps are still available for the current time section, initialised with the value of $a$. The flow chart of the algorithm is illustrated by Figure 6.


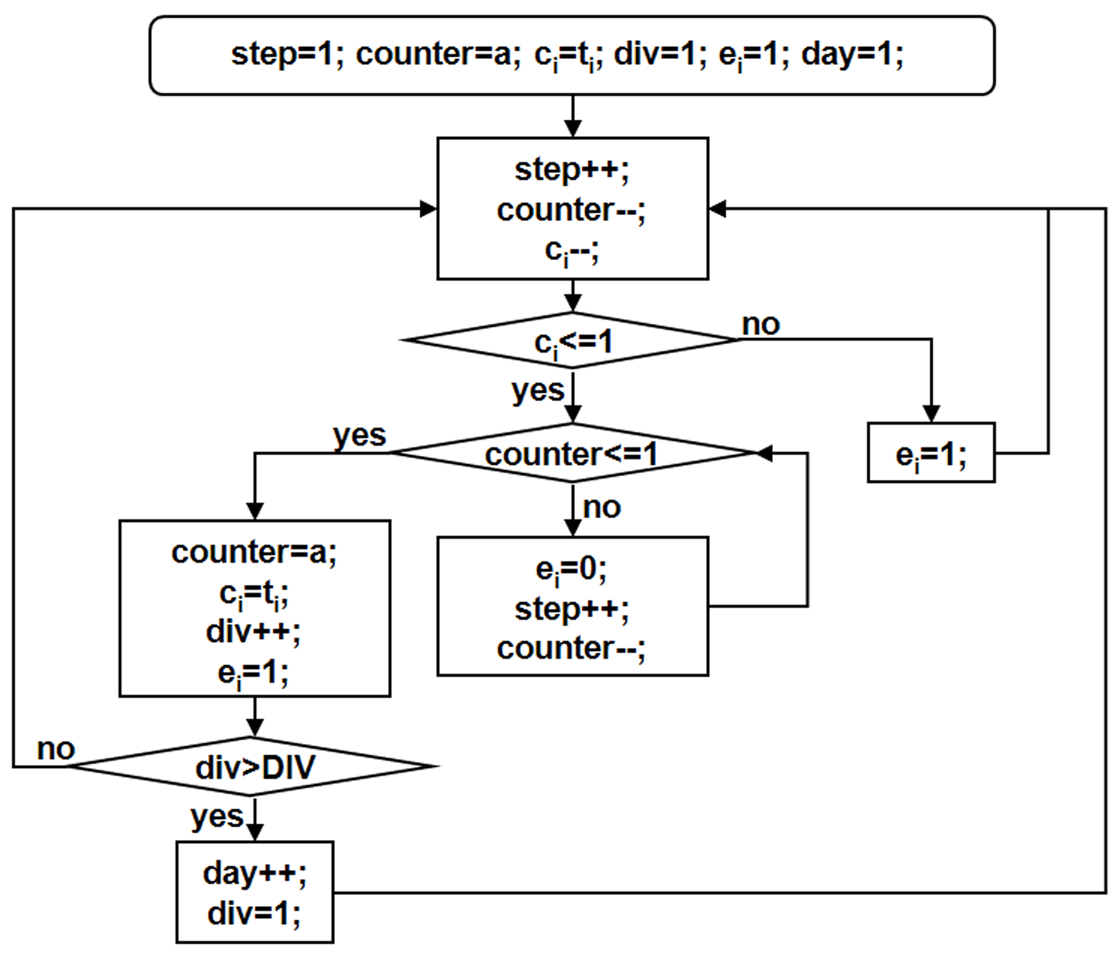


**Figure 6. Synchronisation algorithm for coordination of multi-rate developmental and signalling events.** The values of “counter” and “c_i_” are decremented by 1 after the completion of each L-system time step. When “c_i_” is equal to or lower than 1, its corresponding signalling or development event “e_i_” will be temporarily stopped until the current time section is finished; otherwise the event “e_i_” keeps happening. When “counter” is equal to or lower than 1, a new time section will start and the values of “counter” and “c_i_” will be re-initialised. When the number of time sections (counted by variable “div”) exceeds the value of DIV, a new day will start and the value of “div” will be reinitialised. The expressions in this flow chart are in C syntax.

**References**

1. Room P, Hanan J, Prusinkiewicz P (1996) Virtual plants: new perspectives for ecologists, pathologists and agricultural scientists. Trends Plant Sci 1: 33-38.

2. Han L, Gresshoff PM, Hanan J (2007) Virtual soybean—a computational model for studying autoregulation of nodulation. In: The 5th International Workshop on Functional Structural Plant Models. Napier, New Zealand.

3. Prusinkiewicz P (2004) Art and science for life: Designing and growing virtual plants with L-systems. Acta Hortic 630: 15-28.

4. Prusinkiewicz P, Lindenmayer A (1990) The Algorithmic Beauty of Plants. New York: Springer-Verlag New York, Inc.

5. Renton M, Kaitaniemi P, Hanan J (2005) Functional-structural plant modelling using a combination of architectural analysis, L-systems and a canonical model of function. Ecol Model 184: 277-298.

6. Watanabe T, Hanan JS, Room PM, Hasegawa T, Nakagawa H, et al. (2005) Rice morphogenesis and plant architecture: measurement, specification and the reconstruction of structural development by 3D architectural modelling. Ann Bot-London 95: 1131-1143.
