# Supplementary material for: Computational Complementation: A Modelling Approach to Study Signalling Mechanisms during Legume Autoregulation of Nodulation
Source: PLoS Comput Biol. 2010 Feb 26;6(2):e1000685. doi: 10.1371/journal.pcbi.1000685 (PMC2829028; doi:10.1371/journal.pcbi.1000685)
Supplement: Text S3 — Assumptions and conditions for virtual experiments (0.15 MB DOC) [file pcbi.1000685.s003.doc]

# Text S3: Assumptions and conditions for virtual experiments

Since our testing target for this first application case is the cotyledon-root and the cotyledon-shoot hypotheses, all other signalling mechanisms, including signal production, transport, perception and function, play a supplementary role to support the running of the AON system. The unknown details of the other signalling mechanisms (excluding the testing target) could be temporarily assumed and manipulated according to biologists’ understanding during virtual experiments but were not tested in this case.

**Production of Q**

During early stages of soybean nodulation the bacteria still actively produce nod factor, but expanded nodules with mature bacteroids show no stimulation of the NFR1/5 to CCamK cascade needed for nodulation and induction of SDI [1]. Thus, mature nodules have less SDI stimulating activity and we assume the production of Q signal from each nodulation site to be inversely proportional to nodule development stage, strongest at the stage of nodule initialisation but growing weaker as the nodule matures. Since quantitative knowledge about this process is unclear, we used nodule growth potential to represent this inverse relationship:

(1)

where $Q\text{prdt}$ is the quantity of Q signal produced by a nodulation site at a certain moment, $Q\text{ini}$ is a parameter used to define the relationship with SDI inhibition threshold (see parameter setting below), $N\text{final}$ is the final size of this nodule, and $N\text{current}$ is the current size of this nodule.

**Signal transport**

For the signal transport through roots, stem and petioles, multiple possible patterns of its movement from one tissue to the next are supported by our computational models. For example, the transport could be mass-flow and could also be restricted by certain concentration thresholds. In this case, we assumed mass flow as the transport pattern. The transport rates of Q and SDI were controlled respectively by parameters and $R\text{sdi}$.

**Perception of Q and production of SDI**

The quantitative pattern of Q perception in a leaf is also supported with multiple options. In this application case, we assumed all Q molecules arriving in the leaf could be fully perceived by *GmNARK*. As a consequent event of this perception, the triggered production of SDI at a certain moment was assumed to be proportional to the perceived Q quantity. Since mRNA expression of *GmNARK* apparently is uniform along the leaf vasculature and the vascular content per leaf is proportional to total leaf biomass [2], we used leaf biomass as the coefficient for this proportion relationship:

(2)

Where $\text{SDI}\text{prdt}$ is the quantity of produced SDI signal at a certain moment, $Q\text{pcpt}$ is the perceived quantity right before this event and $B\text{leaf}$ represents the biomass of this leaf at this moment. At a certain moment, the relationship between $\text{SDI}\text{prdt}$ and $Q\text{pcpt}$ is proportional. However, due to the continuous change of leaf biomass, this process is actually nonlinear over time. For unifoliate and trifoliate leaves, their biomass keeps increasing with plant development, thus their capability to produce SDI also kept increasing in this series of virtual experiments. However, the cotyledon biomass declines as the plant grows, thus the production of SDI from the cotyledons during these virtual experiments kept being weakened.

**Function of SDI**

When the SDI signal arrives at a potential nodulation site in the root, a threshold is assumed for determining whether nodule initialisation from this site should be inhibited or not. This threshold is defined as $\text{SDI}\text{ihbt}$ in virtual experiments. If the quantity of SDI signal around a potential nodulation site is higher than $\text{SDI}\text{ihbt}$, the potential nodule will be inhibited; otherwise, the potential nodule will be formed.

**Parameter setting**

The strategy for this application was to adjust parameters for signal production, transport, perception and function within a physiologically appropriate range, as quantitative details about these mechanisms still remain largely unknown. Three qualitative relationships between $Q\text{ini}$ and $\text{SDI}\text{ihbt}$ – namely higher, equal and lower – were represented by setting $Q\text{ini}$ at 1, while varying $\text{SDI}\text{ihbt}$ through values of 0.5, 1 and 2. For signal transport rates, lab experiments demonstrated that auxin, which might play a role in AON, moves at rate of 60 mm/day in soybean (unpublished data), while previous studies suggested that it could be transported “through many plant tissues” at a speed of 240-360 mm/day “in the general direction from the plant’s apex to its roots” [3]. Since SDI might also be other signals and transport rate for Q is unclear, we assumed three values for and $R\text{sdi}$: 60 mm/day, 160 mm/day and 360 mm/day. Combinations allowing different relative speeds for and $R\text{sdi}$ gave 27 different conditions for cotyledon-root testing experiments CRH_1 to CRH_27 and cotyledon-shoot experiments CSH_1 to CSH_27, as shown in Table 1.

**Table 1.** Parameter setting for each virtual experiment.

| **Experiment ID** | **Cotyledon Hypothesis** | **Signal Transport Rates (mm/day)** | | ***Q_ini_ : SDI_ihbt_*** |
| --- | --- | --- | --- | --- |
|  |  | **Q** | **SDI** |  |
| CRH_1 | cotyledon-root | 360 | 360 | 1 |
| CRH_2 | cotyledon-root | 360 | 160 | 1 |
| CRH_3 | cotyledon-root | 160 | 360 | 1 |
| CRH_4 | cotyledon-root | 160 | 160 | 1 |
| CRH_5 | cotyledon-root | 360 | 60 | 1 |
| CRH_6 | cotyledon-root | 60 | 360 | 1 |
| CRH_7 | cotyledon-root | 60 | 60 | 1 |
| CRH_8 | cotyledon-root | 160 | 60 | 1 |
| CRH_9 | cotyledon-root | 60 | 160 | 1 |
| CRH_10 | cotyledon-root | 360 | 360 | 2 |
| CRH_11 | cotyledon-root | 360 | 160 | 2 |
| CRH_12 | cotyledon-root | 160 | 360 | 2 |
| CRH_13 | cotyledon-root | 160 | 160 | 2 |
| CRH_14 | cotyledon-root | 360 | 60 | 2 |
| CRH_15 | cotyledon-root | 60 | 360 | 2 |
| CRH_16 | cotyledon-root | 60 | 60 | 2 |
| CRH_17 | cotyledon-root | 160 | 60 | 2 |
| CRH_18 | cotyledon-root | 60 | 160 | 2 |
| CRH_19 | cotyledon-root | 360 | 360 | 0.5 |
| CRH_20 | cotyledon-root | 360 | 160 | 0.5 |
| CRH_21 | cotyledon-root | 160 | 360 | 0.5 |
| CRH_22 | cotyledon-root | 160 | 160 | 0.5 |
| CRH_23 | cotyledon-root | 360 | 60 | 0.5 |
| CRH_24 | cotyledon-root | 60 | 360 | 0.5 |
| CRH_25 | cotyledon-root | 60 | 60 | 0.5 |
| CRH_26 | cotyledon-root | 160 | 60 | 0.5 |
| CRH_27 | cotyledon-root | 60 | 160 | 0.5 |
| CSH_1 | cotyledon-shoot | 360 | 360 | 1 |
| CSH_2 | cotyledon-shoot | 360 | 160 | 1 |
| CSH_3 | cotyledon-shoot | 160 | 360 | 1 |
| CSH_4 | cotyledon-shoot | 160 | 160 | 1 |
| CSH_5 | cotyledon-shoot | 360 | 60 | 1 |
| CSH_6 | cotyledon-shoot | 60 | 360 | 1 |
| CSH_7 | cotyledon-shoot | 60 | 60 | 1 |
| CSH_8 | cotyledon-shoot | 160 | 60 | 1 |
| CSH_9 | cotyledon-shoot | 60 | 160 | 1 |
| CSH_10 | cotyledon-shoot | 360 | 360 | 2 |
| CSH_11 | cotyledon-shoot | 360 | 160 | 2 |
| CSH_12 | cotyledon-shoot | 160 | 360 | 2 |
| CSH_13 | cotyledon-shoot | 160 | 160 | 2 |
| CSH_14 | cotyledon-shoot | 360 | 60 | 2 |
| CSH_15 | cotyledon-shoot | 60 | 360 | 2 |
| CSH_16 | cotyledon-shoot | 60 | 60 | 2 |
| CSH_17 | cotyledon-shoot | 160 | 60 | 2 |
| CSH_18 | cotyledon-shoot | 60 | 160 | 2 |
| CSH_19 | cotyledon-shoot | 360 | 360 | 0.5 |
| CSH_20 | cotyledon-shoot | 360 | 160 | 0.5 |
| CSH_21 | cotyledon-shoot | 160 | 360 | 0.5 |
| CSH_22 | cotyledon-shoot | 160 | 160 | 0.5 |
| CSH_23 | cotyledon-shoot | 360 | 60 | 0.5 |
| CSH_24 | cotyledon-shoot | 60 | 360 | 0.5 |
| CSH_25 | cotyledon-shoot | 60 | 60 | 0.5 |
| CSH_26 | cotyledon-shoot | 160 | 60 | 0.5 |
| CSH_27 | cotyledon-shoot | 60 | 160 | 0.5 |

**References**

1. Li D, Kinkema M, Gresshoff PM (2009) Autoregulation of nodulation (AON) in *Pisum sativum* (pea) involves signalling events associated with both nodule primordia development and nitrogen fixation. J Plant Physiol 166: 955-967.

2. Nontachaiyapoom S, Scott PT, Men AE, Kinkema M, Schenk PM, et al. (2007) Promoters of orthologous *Glycine max* and *Lotus japonicus* nodulation autoregulation genes interchangeably drive phloem-specific expression in transgenic plants. Mol Plant Microbe In 20: 769–780.

3. Mitchison GJ (1980) The Dynamics of Auxin Transport. P R Soc London 209: 489-511.
