# Supplementary figures and images for: Computational Complementation: A Modelling Approach to Study Signalling Mechanisms during Legume Autoregulation of Nodulation
Source: PLoS Comput Biol. 2010 Feb 26;6(2):e1000685. doi: 10.1371/journal.pcbi.1000685 (PMC2829028; doi:10.1371/journal.pcbi.1000685)

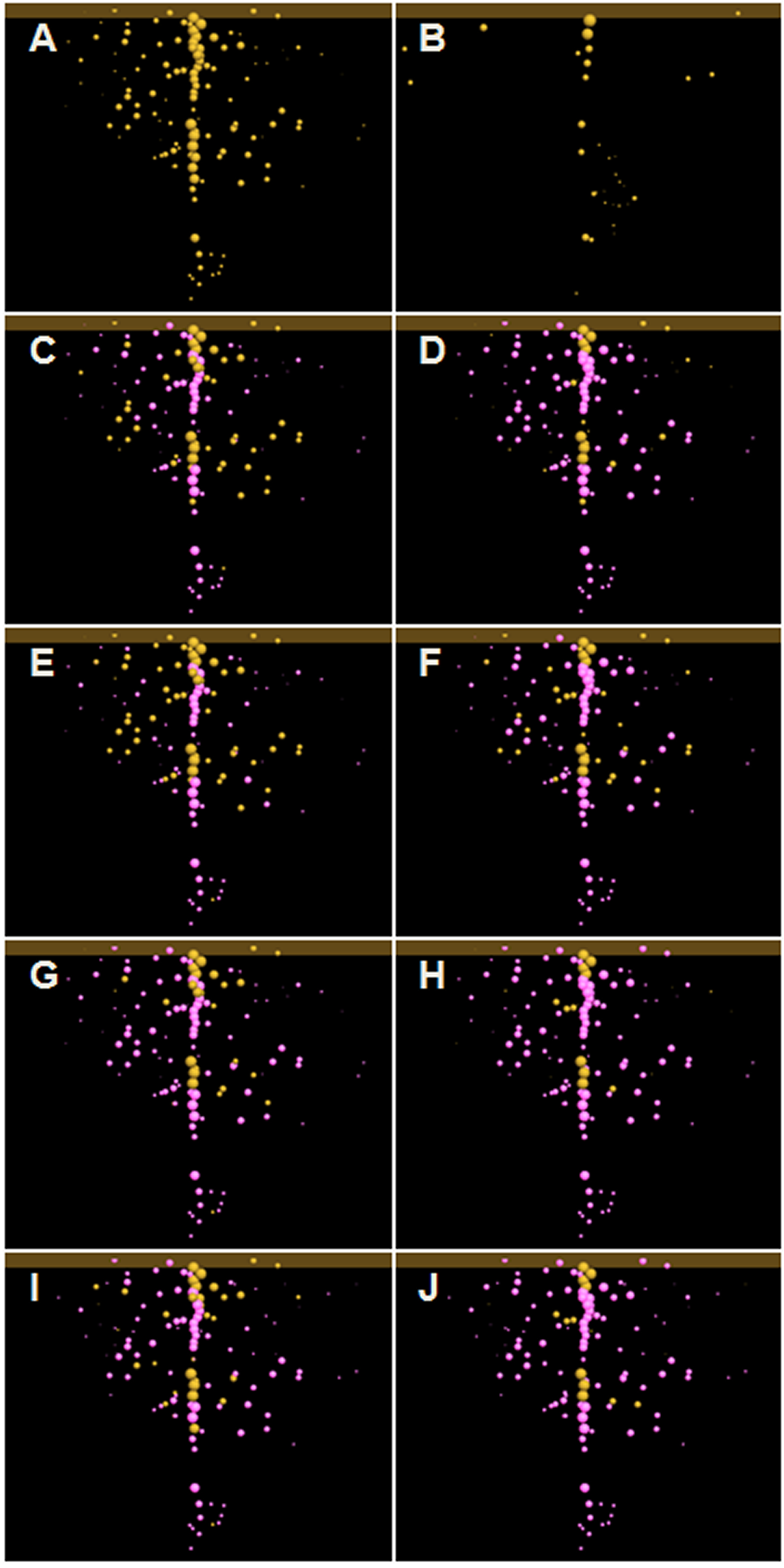

Supplement: Figure S1 — Visualisation of nodule distribution with inhibited nodules on the 16th day post-sowing (2.34 MB TIF) [file pcbi.1000685.s004.tif]
